# A Comparative Analysis of Microbial DNA Preparation Methods for Use With Massive and Branching Coral Growth Forms

**DOI:** 10.3389/fmicb.2018.02146

**Published:** 2018-09-07

**Authors:** Alejandra Hernandez-Agreda, William Leggat, Tracy D. Ainsworth

**Affiliations:** ^1^Australian Research Council Centre of Excellence for Coral Reef Studies, James Cook University, Townsville, QLD, Australia; ^2^College of Public Health, Medical and Veterinary Sciences, James Cook University, Townsville, QLD, Australia; ^3^School of Environmental and Life Sciences, The University of Newcastle, Ourimbah, NSW, Australia; ^4^School of Biological, Earth and Environmental Sciences, University of New South Wales, Sydney, NSW, Australia

**Keywords:** coral microbiome, bacteria, microbial ecology, DESS, paraformaldehyde, snap frozen, bead beating, crushing

## Abstract

In the last two decades, over 100 studies have investigated the structure of the coral microbiome. However, as yet there are no standardized methods applied to sample preservation and preparation, with different studies using distinct methods. There have also been several comparisons made of microbiome data generated across different studies, which have not addressed the influence of the methodology employed over each of the microbiome datasets. Here, we assess three different preservation methods; salt saturated dimethyl sulfoxide (DMSO) – EDTA, snap freezing with liquid nitrogen and 4% paraformaldehyde solution, and two different preparation methodologies; bead beating and crushing, that have been applied to study the coral microbiome. We compare the resultant bacterial assemblage data for two coral growth forms, the massive coral *Goniastrea edwardsi* and the branching coral *Isopora palifera*. We show that microbiome datasets generated from differing preservation and processing protocols are comparable in composition (presence/absence). Significant discrepancies between preservation and homogenization methods are observed in structure (relative abundance), and in the occurrence and dominance of taxa, with rare (low abundance and low occurrence) phylotypes being the most variable fraction of the microbial community. Finally, we provide evidence to support chemical preservation with DMSO as effective as snap freezing samples for generating reliable and robust microbiome datasets. In conclusion, we recommend where possible a standardized preservation and extraction method be taken up by the field to provide the best possible practices for detailed assessments of symbiotic and conserved bacterial associations.

## Introduction

Sequencing of the gene 16S rRNA is now by far the most common technique used to study the microbiome (Shokralla et al., 2012; D’Amore et al., 2016; Lear et al., 2018). The reliability of this method is directly related to the accuracy and precision of capturing entire communities of highly diverse, abundant, and uncultivable microbes (Rajendhran and Gunasekaran, 2011; D’Amore et al., 2016; Thompson et al., 2017). A number of steps are required to undertake this process, starting with the initial sampling protocol, through to the analysis (Lear et al., 2018). Throughout the process of generating a microbiome dataset, the protocol that is used can impact many attributes of the microbial dataset, and consequently, our understanding of the microbial community. Methods that can influence the final dataset may be related to the initial preservation of samples (Vlčková et al., 2012; Gray et al., 2013; Rocha et al., 2014), DNA extraction and amplification (Pinto and Raskin, 2012; Soergel et al., 2012; Ghyselinck et al., 2013), as well as a number of metrics related to downstream sequence analysis (McMurdie and Holmes, 2014).

As preservation methods, three reagents are commonly employed in marine research efforts to identify and characterize the microbiome. Each of these preservation methods has been developed to overcome various limitations of working in remote field sites, where access to fully equipped laboratories is limited (Nagy, 2010). For example, salt saturated dimethyl sulfoxide (DMSO) – EDTA (Seutin et al., 1991) is one of the most widely used preservation methods in marine sampling protocols as it can be transported long distances and remains stable over long time periods (Dawson et al., 1998). Snap freezing has also become widely used as the sample is preserved immediately upon collection with minimal handling and exposure of the sample to preservation artifacts (Fouhy et al., 2015; Vandeputte et al., 2017). However, this method has been limited by the capacity to transport and store liquid nitrogen or dry ice in remote areas. Further, fragments preserved with DMSO or liquid nitrogen are not suitable for histology, a tool that can inform about host health status (as tissue condition, e.g., Ainsworth et al., 2016) and localization of bacteria (e.g., van de Water et al., 2015; Neave et al., 2017) and contributes to identifying the bacterial niche and functional role on coral’s well-being. Fixation with paraformaldehyde (PFA) based solutions (for example, 4% PFA) has recently become more widely used to evaluate the microbiome in plankton, humans, plants, sponges, and corals through flow cytometry and fluorescence *in situ* hybridization (FISH) (Dinsdale et al., 2008; Tang et al., 2011; Lundberg et al., 2012; Raina et al., 2013; Adam et al., 2016; Bruder et al., 2016; Guerrero-Feijóo et al., 2017; Neave et al., 2017). Like DMSO, sample preservation in PFA provides an easily transportable and widely applicable preservation system; but it has not yet been widely taken up in environmental microbiome studies, and the impact of histological preparation on coral microbiome has not been evaluated.

In generating a microbiome dataset, sample homogenization is an essential process within the DNA extraction (Elbrecht and Leese, 2015; Lear et al., 2018). To date, the homogenization processes used in studying the coral holobiont microbiome have varied between studies. In general, some form of crushing of the entire coral sample is employed. Crushing the hard coral skeleton and overlaying tissues involves either the use of a mortar and pestle or a French press whilst the sample is held in liquid nitrogen to prevent DNA degradation (Ng et al., 2015; Samodha et al., 2015; Shore-Maggio et al., 2015; Zhang et al., 2015). Sample lysis and DNA extraction are then applied to a sub-sample of the generated homogenate, for example, using approximately 20 mg of homogenate samples in cell lysis buffer before DNA extraction (e.g., Ainsworth et al., 2015). Homogenization through bead beating of a small sub-sample has also been applied to extraction protocols without the use of prior crushing (e.g., Weber et al., 2017). The bead beating method combines physical force applied on spheres with cell lysis prior to DNA extraction (Lear et al., 2018). This method utilizes a smaller sample (for example, in coral studies ∼1–2 cm of the entire coral branch) and uses the beads to strip the overlaying tissues from the coral skeleton during the chemical cell lysis. The bead beating method is also used to homogenize tissue-mucus slurry airbrushed from coral fragments. This approach provides a quicker and more cost-effective means of sample preparation. Compared to crushing, less of the coral skeleton is being broken down and therefore, may alter the resulting dataset due to less of the endolithic microbiome (microbes contained within the skeleton) being released. There are many advantages and disadvantages to different sample preservation and preparation methods that have been employed in coral, and marine microbiome studies including transport, handling time, handling effort, total cost, and applicability in remote field locations. Despite comparison of DNA extraction kits and homogenization methodologies (Weber et al., 2017), very few studies have directly compared preservation and processing methods to determine their impact on the resulting datasets (e.g., Gray et al., 2013). However, there are studies comparing the microbiome datasets generated from multiple studies (Mouchka et al., 2010; Miller and Richardson, 2011).

Assessing preservation and homogenization methods can provide insights into protocols best suited for use in remote locations and assist in standardizing approaches across different studies undertaken worldwide. Standardized protocols are particularly relevant for microbiome studies on coral reefs. The worldwide degradation of coral reef ecosystems is driving more and more studies to be undertaken on the coral microbiome. Studies are aiming to define the characteristics of microbial communities of healthy organisms and also dysbiotic and unhealthy coral reef ecosystems (Ainsworth and Gates, 2016; Bourne et al., 2016). Coral reef ecosystems are often remote and located offshore, and sampling undertaken in these areas often represents a compromise in the number of samples taken and the quality of the preservation method. These logistical constraints are acknowledged as potential influencing factors on the microbiome datasets that are generated and consequently, on our perception of the attributes of microbial communities. The current study aims to evaluate the influence of three sample preservation methods and two DNA extraction protocols on the microbiome datasets generated from two coral species.

## Materials and Methods

### Coral Collection and Preservation

**FIGURE 1:**
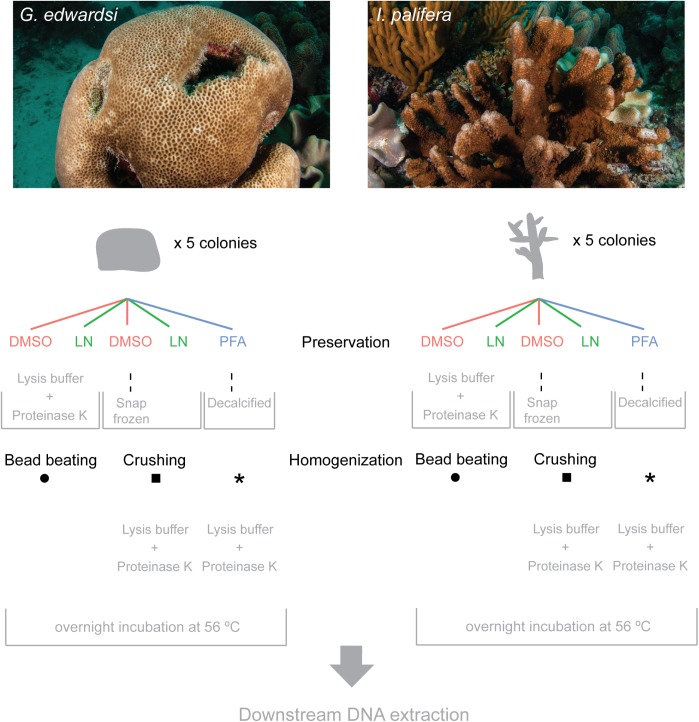
Flow diagram of experimental design. Five fragments were collected per colony; two were preserved in DMSO, two in LN and one in PFA. PFA-preserved fragment was decalcified before DNA extraction. One fragment per each preservation method was homogenized using either bead beading or crushing method. LN, liquid nitrogen; DMSO, salt saturated dimethyl sulfoxide (DMSO) – EDTA; PFA, paraformaldehyde. Photos by Ed Roberts.

On January 2015, fragments of corals *Goniastrea edwardsi* (*n* = 25, <3 cm diameter) and *Isopora palifera* (*n* = 25, <5 cm long) were collected from the reef flat at Coral Gardens reef adjacent to Heron Island Research Station, Australia (23°26.5248′ S, 151°54.754′ E). For each species five coral fragments were collected from five colonies separated by >3 m, using a hammer and chisel (**Figure 1**). After collection, the samples were held in seawater and immediately preserved (within the first 2 h). For each colony, samples were preserved using three reagents: two samples were snap frozen in liquid nitrogen and stored at -80°C, two samples were preserved in salt-saturated 20% dimethyl sulfoxide (DMSO) – 0.5 M EDTA and stored at 4°C, and one sample was fixed in 4% PFA solution and stored at 4°C. After 14 h samples fixed in PFA were rinsed and stored in sterile 3x phosphate buffered saline at 4°C. 4% paraformaldehyde solution and 3x phosphate buffered saline were prepared using DNA/RNA-free water on the same day of coral collection. Fragments were shipped to James Cook University, Townsville, QLD, Australia. Until their processing, fragments preserved in PFA and DMSO were stored at 4°C, and snap frozen fragments at -80°C. Coral fragments were collected under permits supplied by the Great Barrier Reef Marine Park Authority (Townsville, QLD, Australia, G15/37488.1).

### Sample Homogenization and Decalcification

For a mixed combination preservation reagent × homogenization method, samples preserved in liquid nitrogen and DMSO were homogenized using two methods, bead beating and crushing (**Figure 1**). To standardize the sample size, a subsample of 0.173 ( ± 0.04) g of coral, including tissue and skeleton, were used for both methods. Homogenization under liquid nitrogen is necessary to ensure a uniform homogenization across the entire sample, thus colony samples preserved in DMSO were snap frozen before crushing. As such, one sample preserved in liquid nitrogen and one in DMSO (after being snap frozen) were crushed in liquid nitrogen applying up to 40 psi of pressure with a French press, followed by manual homogenization to a fine powder using mortar and pestle on dry ice (**Figure 1**). The resulting powder was used for subsequent steps in cell lysis and DNA extraction. All instruments were sterilized prior to use with each sample. The resulting homogenate was used in the DNA extraction outlined below (see section “DNA Extraction, Amplification, and Sequencing Protocol”). For homogenization using bead beating, the same amount of coral tissue/skeleton from each sample preserved in DMSO and liquid nitrogen were individually placed into 2 ml tubes with 1.0 mm silica spheres for immediate lysis and DNA extraction. 360 μl of lysis buffer (QIAmp^®^ DNA Mini Extraction kit, Qiagen) and 40 μl of Proteinase K were added to each tube. A FastPrep-24^TM^ 5G (MP) homogenizer was used to run three rounds of 20 s each at 4.0 m/s to homogenize the sample.

Samples preserved in PFA were decalcified before DNA extraction to evaluate the viability of the use of a preservation method that allows both taxonomic profiling and histological evaluation. For each sample preserved in PFA and stored in PBS, the entire coral sample was decalcified with repetitive washes of DNA/RNA-free 20% EDTA at 4°C over a 2-week period. After decalcification of the entire coral sample, 0.04 ( ± 0.004) g of the resulting coral tissue was used from each colony for successive steps in DNA extraction.

### DNA Extraction, Amplification, and Sequencing Protocol

Tissue from the decalcified samples and the powder from crushed samples were individually placed in 1.5 ml tubes. 360 μl of lysis buffer (QIAmp^®^ DNA Mini Extraction kit, QIAGEN) and 40 μl of Proteinase K were added to each tube.

Together with homogenized samples from the bead beating method, all samples were incubated overnight at 56°C and posteriorly purified using a silica-membrane-based nucleic acid technique as per the manufacturer’s protocol (QIAmp^®^ DNA Mini Extraction kit, QIAGEN). Extracted DNA concentration and purity were quantified using Qubit Fluorometer and Qubit^®^ dsDNA High-sensitivity Assay Kit (Life Technologies, Thornton, NSW, Australia). Extracted DNA was stored at -20°C before PCR amplification and sequencing. DNA extraction, amplification, and sequencing were performed on negative controls (no sample template) as well.

Genomic template primers 27F/519R (v1–v3 region) and barcode on the forward primer were used in a 30-cycle PCR using HotStarTaq plus master mix kit (QIAGEN, United States) to amplify bacterial 16S rRNA gene amplicons. PCRs were run under following conditions: 94°C for 3 min, followed by 28 cycles of 94°C for 30 s, 53°C for 40 s, and 72°C for 1 min, a final elongation at 72°C for 5 min. Based on molecular weight and DNA concentration, amplicon products from different samples were pooled and purified using calibrated Ampured XP beads. DNA libraries were prepared with purified and pooled samples following the Illumina TruSeq DNA library preparation protocol. Sequencing was performed at MR DNA (Shallowater, TX, United States) on a MiSeq platform following manufacturer’s protocol. 16S rRNA raw sequences are available in the National Center for Biotechnology Information (NCBI) Short Read Archive (SRA) under the Project No. PRJNA432131, Accession Nos. SAMN08442327 to SAMN08442375.

### Sequence Analysis

Sequence data were processed using the open-source software Quantitative Insights Into Microbial Ecology (QIIME, version 1.9) (Caporaso et al., 2010). Barcodes, ambiguous base calls, and homopolymer runs exceeding 6 bp were removed from raw sequence data. Only sequences with a minimum quality score of 25 and length 200–1000 bp were used in the analysis (278,089 sequences discarded). Chimeras sequences were removed using Usearch61 (Edgar et al., 2011). Sequences were clustered with cd-hit (Li and Godzik, 2006) at 97% similarity to define operational taxonomic units (OTUs). RDP classifier (Wang et al., 2007) was used against a curated Greengenes database (version 13_8) (DeSantis et al., 2006) to assign taxonomy to OTUs. Chloroplast, mitochondria, unidentified, and unassigned OTUs were removed from resulting OTU tables.

### Statistical Analyses

Differences between preservation and homogenization methods were analyzed using PRIMER v7 and PERMANOVA+ (Anderson et al., 2008). The overall performance of each methodology was assessed through the comparison of the number of OTUs, richness (Margalef’s index, *d*), diversity (Shannon–Wiener, *H′*), evenness (Pielou’s evenness, *J′*), taxonomic breadth [Average (Δ^+^) and Variation (Λ^+^) of taxonomic distinctness], and bacterial assemblage composition and structure. As part of a comprehensive evaluation of the different preservation and homogenization methods, singletons, and low read OTUs were kept in the data analysis. For the analysis of relative abundance, a fourth root transformation and standardization by sample by total was applied to the OTU table (McMurdie and Holmes, 2014). The OTU table was also converted to presence/absence to evaluate bacterial composition. Differences between methodologies were evaluated with a design considering both *Preservation* and *Homogenization* as fixed factors with two levels each (DMSO and liquid nitrogen; and bead beating and crushing, respectively). Individual comparisons between decalcified PFA fixed samples and other samples under preservation-homogenization combinations were assessed with a design considering the combination preservation-homogenization as *Treatments*, a fixed factor with five levels (DMSO-BB, DMSO-Cr, LN-BB, LN-Cr, and PFA-decalcified).

Differences between preservation and homogenization methods and treatments were identified by permutational multivariate analysis of variance (PERMANOVA) on Euclidian distances (diversity metrics), Bray–Curtis (BC) and Sorensen dissimilarity matrices (relative abundance and presence/absence data, respectively). PERMANOVA analyses were run under the following parameters: type III (partial) sums of squares, fixed effects sum to zero for mixed terms, number of permutations 9,999 and as permutation method, permutation of residuals under a reduced model for the assessment of differences between preservation and homogenization methods, and unrestricted permutation of raw data for analysis of differences between treatments. Adjusted Bonferroni *p-value* was used to determine significant differences between PFA fixed samples and other samples under preservation-homogenization combinations. Coral species data were analyzed separately since differences between them were detected (**Supplementary Figure 1** and **Supplementary Tables 3, 4**). Two-dimensional non-metric dimensional scaling (nMDS) plots (Clarke, 1993) are presented to illustrate PERMANOVA results.

The OTUs present across samples of a treatment (core 100% per treatment) were determined using the command *compute_core_microbiome.py* in QIIME. Venn diagrams were generated using the Venn diagram software (Bioinformatics and Evolutionary Genomics^1^). Graphs were produced using ‘ggplot2’ package in R Core Team (2013) and Wickham (2016).

## Results

### Number of Sequences, Operational Taxonomic Units (OTUs), and Diversity Metrics

**FIGURE 2:**
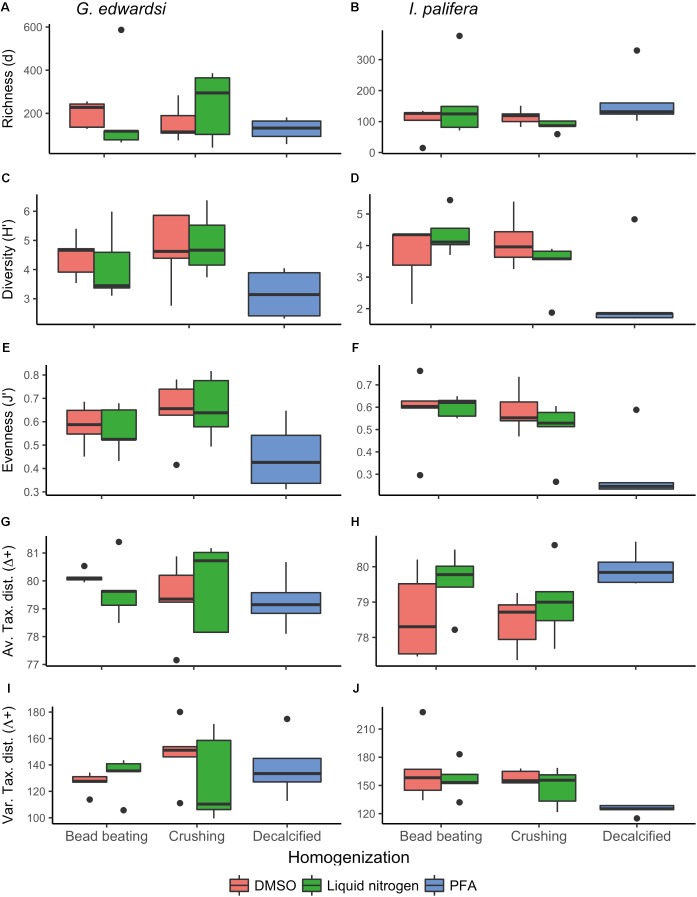
Diversity metrics for *Goniastrea edwardsi* **(A,C,E,G,I)** and *Isopora palifera* **(B,D,F,H,J)** microbiome. No significant differences were detected in the diversity metrics of *G. edwardsi* microbiome (**Supplementary Table 1**). *I. palifera* microbiome showed differences between fragments preserved with PFA and DMSOCr in evenness, average, and variation of taxonomic distinctness, and LNBB in evenness and variation of taxonomic distinctness (PERMANOVA, **Supplementary Table 2**). Richness (d): Margalef’s index, Diversity (H′): Shannon diversity, Evenness (J′): Pielou’s evenness, Av. Tax. dist. (Δ^+^): Average of taxonomic distinctness, Var. Tax. dist. (Λ^+^): Variation of taxonomic distinctness. Boxplots are based on raw data after excluding chloroplast, mitochondria, unidentified, and unassigned OTUs.

The number of sequences and the number of OTUs generated was highly variable within all the replicates and between the treatments (**Table 1**), and negative controls did not amplify and did not generate sequences. For the *G. edwardsi* microbiome, on average all of the preservation methods resulted in between 42 and 47 thousand sequences, notably the combination of DMSO-crushing resulted in on average only 22 thousand sequences (**Table 1**). On average, the number of OTUs generated was between 1,350 and 2,527. Notably, the PFA-decalcification method retrieved comparable results to the other methods for both the number of sequences and the number of generated OTUs. Richness (*d*), diversity (*H′*), and evenness (*J′*) of the *G. edwardsi* microbiome were similar for the combination preservation (DMSO and liquid nitrogen) and homogenization method (bead beating and crushing) (**Figures 2A,C,E**). Microbial assemblages from PFA-preserved *G. edwardsi* showed similar richness, but lower diversity and evenness. Taxonomic breadth expressed as average and variation of taxonomic distinctness (Δ^+^ and Λ^+^, respectively), were comparable among all preservation and homogenization methods; however, it was more variable for the treatment liquid nitrogen-crushing (**Figures 2G,I**). As such, there was no significant difference detected between preservation or homogenization methods in the diversity metrics evaluated in the *G. edwardsi* microbiome (**Supplementary Table 1**).

**Table 1:** Number of sequences and OTUs per treatment.

Coral species	Method		N. samples	N. sequences (total)	N. sequences (av. by samples)	N. OTUs (total)	N. OTUs (av. by samples)
						
	Preservation	Homogenization					
*G. edwardsi*	DMSO	Bead beating	5	235,005	47,001	9,522	2,134
	DMSO	Crushing	5	110,634	22,127	6,823	1,530
	Liquid nitrogen	Bead beating	5	212,459	42,492	9,658	2,117
	Liquid nitrogen	Crushing	5	211,469	42,294	10,790	2,527
	PFA	Decalcified	4	190,768	47,692	4,824	1,350
*I. palifera*	DMSO	Bead beating	5	186,429	37,286	4,612	1,071
	DMSO	Crushing	5	140,986	28,197	4,979	1,182
	Liquid nitrogen	Bead beating	5	211,050	42,210	7,787	1,740
	Liquid nitrogen	Crushing	5	146,383	29,277	3,647	890
	PFA	Decalcified	5	476,036	95,207	7,769	1,936


*Counts are estimated on raw data after filtering out chloroplast, mitochondria, unidentified, and unassigned OTUs.*

In *I. palifera*, on average the lowest number of sequences were retrieved from the crushing protocol (28–29 thousand of sequences), followed by for both preservation methods when bead beading (37–42 thousand of sequences), and PFA with the highest value, doubling and tripling the value observed with other methods (95 thousand of sequences, **Table 1**). All preservation and homogenization methods retrieved similar richness (**Figure 2B** and **Supplementary Table 2**). Microbial assemblages from PFA-preserved *I. palifera* showed lower diversity and evenness, although significant differences were only detected in evenness between DMSO-crushing and LN-bead beating, and PFA (**Figures 2D,F** and **Supplementary Table 2**). DMSO-preserved individuals presented a lower average of taxonomic distinctness (Δ^+^) than in liquid nitrogen-preserved and PFA-preserved individuals (**Figure 2H**). PFA-decalcified individuals showed a lower variation of taxonomic distinctness (Λ^+^) than all the other combinations of preservation and homogenization methods (**Figure 2J**). However, significant differences were only detected in the average of taxonomic distinctness (Δ^+^) between DMSO-crushing and PFA, and in the variation of taxonomic distinctness (Λ^+^) between DMSO-bead beating, DMSO-crushing and LN-bead beating, and PFA.

### Community Composition and Structure

**FIGURE 3:**
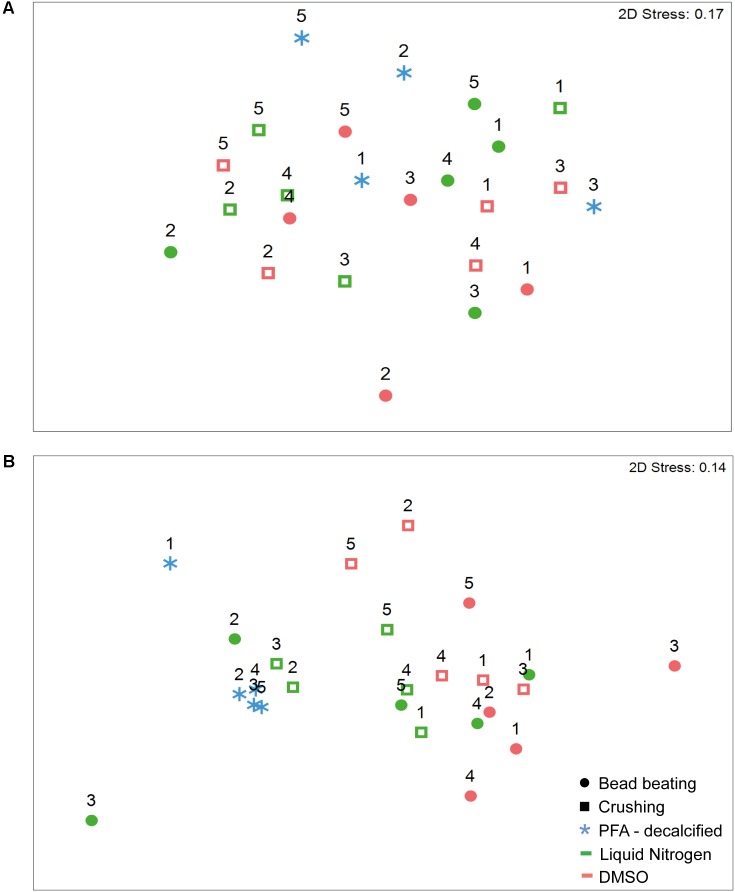
nMDS ordination of bacterial assemblages’ relative abundance in *G. edwardsi* **(A)** and *I. palifera* **(B).** Bacterial communities are similar regardless of the preservation and homogenization method used in *G. edwardsi* bacterial assemblages **(A)**. *I. palifera* **(B)** bacterial assemblages treated with PFA-decalcified differ from the other methods. nMDS based on Bray–Curtis dissimilarity of relative abundance data (fourth root transformed). Colonies indicated with numbers. For presence/absence equivalent results see **Supplementary Figure 2** and **Supplementary Tables 6, 8, 10, 12**.

An analysis of the community structure indicated differences in composition and structure between both coral species (**Supplementary Figure 1** and **Supplementary Tables 3, 4**). Exploring the coral species separately, we found there were no significant differences for either composition or structure of the community retrieved from preservation with DMSO and liquid nitrogen and homogenization using bead beating and crushing methods. In the massive coral *G. edwardsi* bacterial community only 7% of the variation resulted from preservation methods (**Figure 3A** and **Supplementary Figure 2A, Supplementary Tables 5, 6**). We also found there were no evident differences between PFA-decalcification bacterial community composition and structure and the community structure of other methods (**Supplementary Tables 7, 8**). Similarly, for *I. palifera*, no differences were detected between DMSO and liquid nitrogen preservation and bead beating and crushing homogenization. 13 and 9% of variation were assigned to preservation and homogenization methods, respectively (**Figure 3B**, **Supplementary Figure 2B**, and **Supplementary Tables 9, 10**). Contrary to the observed in *G. edwardsi*, bacterial community composition and structure of PFA-decalcified individuals in *I. palifera* were different to the community in individuals preserved with DMSO, regardless the homogenization method (DMSO – Bead beating and crushing in **Figure 3B** and **Supplementary Figure 2B, Supplementary Tables 11, 12**).

### Rare, Common, and Core Microbiome

**FIGURE 4:**
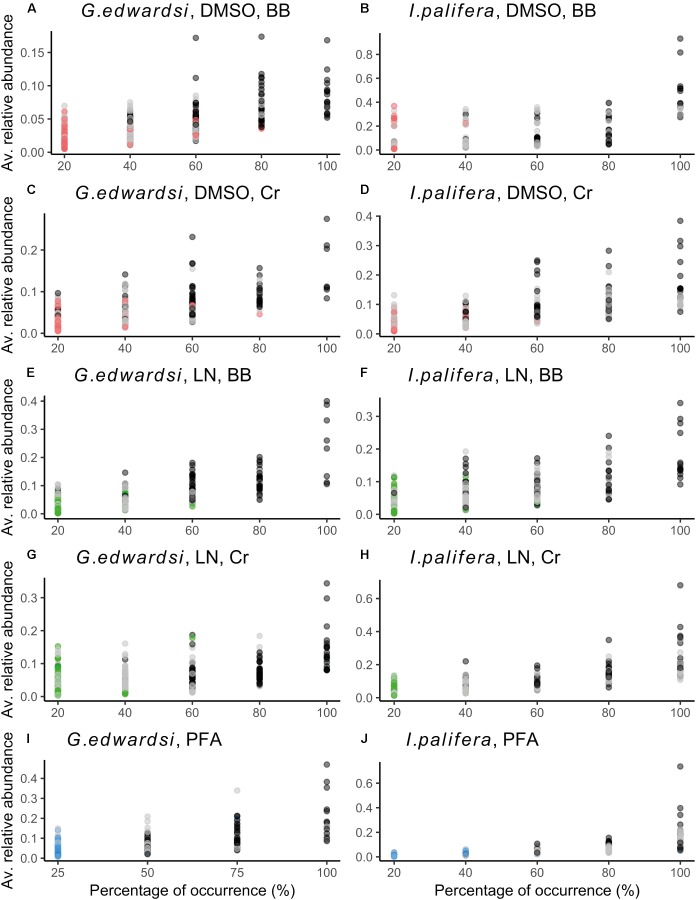
Common/shared and specific phylotypes in bacterial assemblages sampled by different preservation and homogenization methods in *G. edwardsi* **(A,C,E,G,I)** and *I. palifera* **(B,D,F,H,J)**. Graphs of the average relative abundance vs. percentage of occurrence across methodologies revealed that specific bacterial phylotypes for each method are rare, low occurrence and low abundance (red, green, and blue dots across figures). Phylotypes with high occurrence and dominance, are common/shared OTUs across methodologies (gray and black dots). Red, green, and blue dots: OTUs present uniquely in the assemblage sampled by the referred combination method; gray dots: OTUs present in between 2 and 4 of the methods used; black dots: OTUs present in all the methods used. Left side: *G. edwardsi*, right side: *I. palifera*. Green: liquid nitrogen, red: DMSO, blue: PFA. LN, liquid nitrogen; BB, bead beating; Cr, crushing.

We found that bacterial phylotypes with high occurrence and high abundance were captured by all the preparation protocols used in the current study (black dots in **Figure 4**). Interestingly in each methodology, a specific group of bacteria only occurred in between one to three individuals and in low abundance (bright colored dots in **Figure 4**). Differences between the preservation and homogenization methods occured in a fraction of the community that is rare, e.g., bacteria showing low abundance and low occurrence.

**FIGURE 5:**
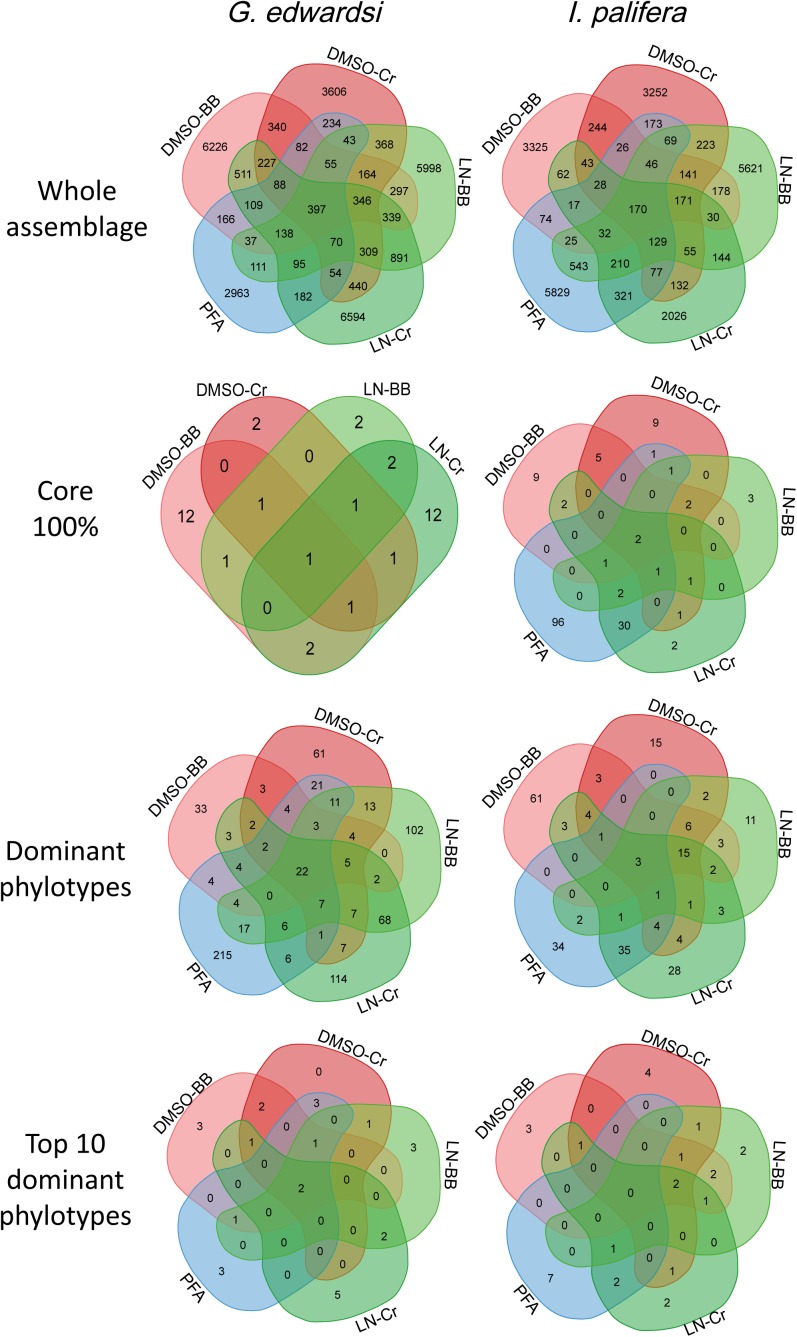
Venn diagrams for the whole bacterial community, core 100%, dominant phylotypes and top 10 dominant phylotypes. Venn diagrams reflect that approximately 39 and 45% of OTUs of the whole bacterial assemblages in *G. edwardsi* and *I. palifera* are shared among methodologies. However, shared bacterial phylotypes are not consistently present as core microbiome, dominant or among the top 10 dominant OTUs across methodologies. Conversely, the ‘importance’ of bacterial phylotype, expressed as occurrence or relative abundance, varies among preservation and homogenization methods (**Supplementary Figures 3, 4** and **Supplementary Table 13**). For OTUs taxonomic identification in core 100%, dominant phylotypes and top 10 dominant phylotypes see **Supplementary Tables 14, 15**.

Dissecting the number of OTUs by their percentage of occurrence demonstrated similar performance between the methods assessed (**Supplementary Figures 3, 4** and **Supplementary Table 13**). Across the methodologies, singletons represented 60% of the total of phylotypes, and while increasing the occurrence, the number of OTUs decrease within the same order of magnitude. Each methodology captured different bacterial communities (**Figure 5**); in the sense that phylotypes showing high occurrence (core 100%) and abundance (dominant OTUs based on the cut-off, and top 10 dominant phylotypes) differed between methodologies (**Supplementary Tables 14, 15**). However, some taxa were consistently detected in all the methodologies with the same dominance or occurrence. For example, for both *G. edwardsi* and *I. palifera* core 100%, OTUs from the family Endozoicimonaceae (except DSMO-BB in *I. palifera*) and genera, *Diaphorobacter* and *Propionibacterium* were detected in all the methodologies employed. OTUs from the Order Kiloniellales, Families Aerococcaceae, Endozoicimonaceae, Flammeovirgaceae, Phyllobacteriaceae, Rhodobacteraceae, and genera *Corynebacterium, Diaphorobacter*, SGUS912, *Propionibacterium*, and *Pseudomonas* were dominant across methodologies for *G. edwardsi* bacterial community. In *I. palifera*, OTUs from the Family Aerococcaceae were consistently found as dominant in the bacterial community (**Supplementary Tables 14, 15**).

### Taxonomic Composition and Structure

**FIGURE 6:**
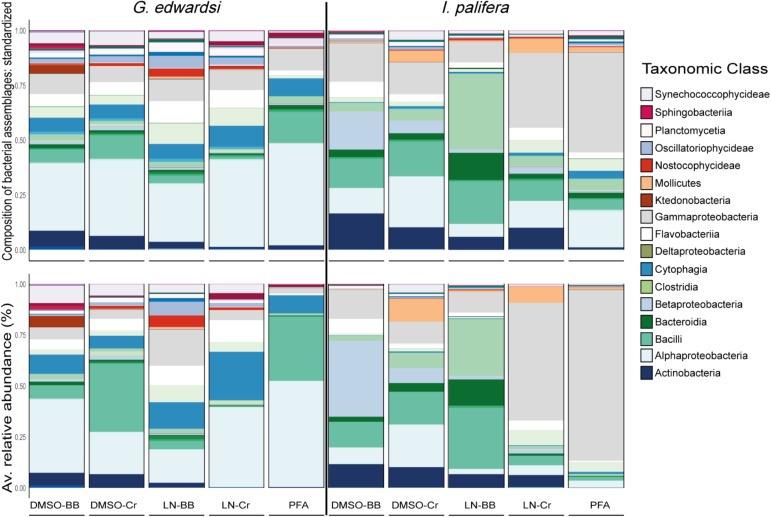
Bacterial assemblage composition (top) and structure (bottom) among preservation and homogenization methods in *G. edwardsi* (left) and *I. palifera* (right). The taxonomic composition is consistent among methodologies for *G. edwardsi* (top left); however, the taxonomic structure is distorted when evaluated using relative abundance (bottom left). For *I. palifera*, the taxonomic composition is not consistent across methodologies, with major discrepancies in classes Gammaproteobacteria, Clostridia, Betaproteobacteria, Bacilli, Alphaproteobacteria, and Actinobacteria (top right). Discrepancies are enhanced when relative abundance is considered (bottom right). For results by colony see **Supplementary Figure 5**. Major taxonomic classes are presented in the legend, for complete legend see **Supplementary Figure 6**.

The taxonomic composition and structure were similar across methodologies for both species (**Figure 6** and **Supplementary Tables 1, 2**); however, for *I. palifera* some of the classes were overrepresented. Consistently high numbers of bacterial phylotypes belonging to classes Alphaproteobacteria, Cytophagia, Flavobacteriia, and Gammaproteobacteria, were evident in *G. edwardsi*. However, small differences occurred in low occurrence classes as Actinobacteria, Sphingobacteriia, and Synechococcophycideae. For *I. palifera*, bacterial classes with the higher number of OTUs were less evident across methodologies. For colonies preserved in DMSO, classes with the higher number of bacterial phylotypes were Gammaproteobacteria and Bacilli, but differences were raised between homogenization methods for classes Alpha-, Beta-proteobacteria, and Actinobacteria. High similarity was evident between liquid nitrogen-crushing, and PFA treated colonies, where Gammaproteobacteria and Alphaproteobacteria were the groups with the higher number of OTUs, whereas LN-BB had an overall distinct taxonomic representation with Clostridia as the class with the higher percentage in composition. As expected, variability between colonies was evident. However, representation of taxonomic composition per colony was similar across methodologies (Top **Supplementary Figure 5**).

The taxonomic structure observed in *G. edwardsi* was less evident in most of the treatments when evaluating relative abundance of the same classes (**Figure 6** and **Supplementary Figure 5** bottom), and differences between methods observed in *I. palifera* were enhanced. In relative abundance, the dominance of the classes Alphaproteobacteria, Cytophagia, and Gammaproteobacteria were still evident in *G. edwardsi* individuals homogenized using bead beating, regardless of the preserving method. Increases in the dominance of Bacilli and Cytophagia were evident when homogenizing with the crushing method, regardless of the preserving method. Alphaproteobacteria, Bacilli, and Cytophagia dominated PFA taxonomic structure; the representation of Gammaproteobacteria was smaller. For *I. palifera*, dominant groups in taxonomic composition had the higher percentages of relative abundance. Alpha-, Betaproteobacteria, Bacilli, and Clostridia dominated DMSO-BB, DMSO-Cr, and LN-BB, however, Gammaproteobacteria still appeared as the second most dominant class. LN-Cr and PFA showed a similar community but were very different from the other methodologies, with the dominance of Gammaproteobacteria, and other groups with lower relative abundance. The contrast between the taxonomic structure and the relative abundance evidenced incongruences observed when comparing 10% and the 10 top most abundant OTUs between different methodologies (**Figure 5**).

## Discussion

Here, we show that sample preservation and processing methodologies generate coral microbiome databases similar in composition, but with structural discrepancies. We find that there is substantial variability in the microbiome between colonies, regardless of the preparation method utilized and this within individual variability is greater than variability resulting from the preparation method employed. No statistical differences are detected in the diversity metrics or community composition and structure of the *G. edwardsi* microbiome. *I. palifera* microbiome is similar and comparable in richness but showed differences in diversity, evenness and taxonomic breadth. Similarly, across methodologies, the same taxonomic classes were retrieved, and there are groups of high occurrences and dominant phylotypes consistently detected. However, there are some evident differences in the percentages of representation of the phylotypes across methodologies. Rare – low abundance bacterial phylotypes represent a high percentage of the assemblage and are specific per preservation-homogenization method. As a result, groups of phylotypes identified as rare – low abundance, dominant and with high occurrence vary between methodologies. Taken together these results indicate that each methodology is sensitive to specific groups of bacteria. Variations in relative abundance and occurrence of shared bacterial phylotypes across methods indicate that both parameters should be considered in conjunction where studies aim to determine the complexity of bacterial communities and to select core phylotypes of interest (i.e., ubiquitous bacterial phylotypes).

If the objective is to evaluate the microbiome composition, the two most widely utilized preservation protocols, DMSO, and liquid nitrogen, coupled with homogenization through either bead beating and crushing methods are directly comparable. The microbiome dataset generated through PFA preservation methods is similar to that of other methods depending on the coral growth form or species. We show that preservation with PFA is directly comparable with the other preservation-homogenization methods for *G. edwardsi* bacterial assemblages. For example, in *G. edwardsi* PFA fixation generates a bacterial assemblage with attributes similar to the assemblages retrieved from other methodologies. The number of sequences and OTUs are in the same order of magnitude, diversity metrics are similar, and the community structure is comparable. For *I. palifera* bacterial assemblages, PFA treated colonies are comparable to those preserved with liquid nitrogen. However, bacterial assemblage retrieved from individuals of *I. palifera* preserved with PFA is different from that of those preserved in DMSO. Diversity, and evenness are in fact lower in PFA preserved samples than the other methodologies. Also, the community structure of PFA preserved individuals seems to be more similar across the coral colonies, with less variation between individuals. In *I. palifera*, we also show that the taxonomic structure of individuals preserved with PFA is similar to those preserved in liquid nitrogen and crushed, and PFA shows similar results in relative abundance vs. percentage of occurrence. Thus, bacterial phylotypes with high occurrence and abundance (see top 10 dominant phylotypes, **Supplementary Table 15C**) are also present in PFA detected bacterial assemblage, and as observed in assemblages treated with other methods, OTUs specific for this method of preservation are present as rare members of the assemblage.

Selecting preservation and homogenization methodology can be influenced by the logistics of the sampling effort without greatly impacting the composition of the microbiome dataset generated. The methods explored in the current study present diverse advantages regarding safety, practicality, reproducibility, and risk of cross-contamination that must be considered when selecting preservation and homogenization methods (Nagy, 2010). For example, DMSO requires handling of dangerous chemicals and training in the preparation of the reagents, which can be a limiting factor for monitoring programs using sampling protocols conducted in association with volunteer groups, but it is a stable preservative in the long term, and no refilling or handling is required after sample collection. Preservation with DMSO can be done in the field at room temperature, and once in the final destination, samples can be stored at -20°C to avoid DMSO evaporation. Therefore, sample refrigeration is not necessary for the short-term when preserving with DMSO. Preservation in PFA is a similarly fast and easy method in the field but requires further handling after sample collection for storage of the sample in phosphate buffered saline (non-hazardous) to avoid over-fixation of the sample. PFA is also hazardous, and handling requires training and safety equipment, with similar limitation for untrained personnel. The advantage of PFA over the other methods is that it is ideal to preserve tissue structure and allows a more detailed assessment of the health and condition of the samples collected, allowing for histological analysis to be conducted, and the identification of bacteria niches through FISH (e.g., Bythell et al., 2002; Ainsworth et al., 2006, 2015; Apprill et al., 2009, 2012). Liquid nitrogen, however, is currently the most common method of preservation for analysis of both DNA and RNA. However, access to liquid nitrogen and -80°C freezers are limited in remote areas, and the transport of liquid nitrogen is prohibited in planes and boats. Preservation with liquid nitrogen also presents a disadvantage for the shipment of samples in specialist dewars, which is time sensitive and expensive (Nagy, 2010). Logistical considerations such as these are likely to impact the preferred method of preservation for any given study of the microbiome in coral and other marine organism samples.

In selecting appropriate methodologies for generating microbiome datasets, it is essential to consider that there are steps in the DNA extraction process that can potentially produce cross-contamination and the homogenization protocol used is one critical consideration. In this study, negative controls (no template) were used in the DNA extraction, amplification, and sequencing, under the same conditions as other samples. These controls did not produce sequences; herein it is not possible to estimate which method is more susceptible to cross-contamination. However, based on the characteristics of the methodologies, we argue that the bead beating is less susceptible. Bead beating is highly reproducible and practical as the homogenization is carried out by a programmed machine and 24 samples can be homogenized at a time. Therefore, the risk of cross-contamination between samples is low because there is little overlap in the handling of the samples (Lear et al., 2018). Crushing samples with a French press or mortar/pestle is a widely employed homogenization method in the study of the coral holobiont microbiome (Ng et al., 2015; Samodha et al., 2015; Shore-Maggio et al., 2015; Zhang et al., 2015). However, reproducibility is questionable since the homogenization with mortar and pestle is manual, variable between samples and the number of samples to homogenize is dependent on the capacity to clean and sterilize the instruments and keep them frozen during the process to prevent the sample becoming mucus bound at room temperature. As such, the risk of cross-contamination is high, because the material is in contact with laboratory instruments and open to the environment while homogenization with mortar and pestle is carried out. Thus, we recommend that future studies apply a bead beating approach to sample preparation rather than sample crushing. Further, genera *Propionibacterium, Corynebacterium, Staphylococcus*, and *Pseudomonas* were found as members of core microbiome or as dominant phylotypes. These genera have been detected as contaminants from reagents and the laboratory environment (Salter et al., 2014). The lack of results in negative controls, and previous evidence of other genus identified as contaminants (*Ralstonia*, Salter et al., 2014) within coral and zooxanthellae cells (Ainsworth et al., 2015), suggest that the relevance of these genera (as contaminants or consistent member of the community) should be tested in corals.

## Conclusion

Our results indicate that comparisons of 16S rRNA databases across different preservation and homogenization methods should be restricted to overall microbiome composition. Important variations were observed in criteria based on the occurrence and relative abundance of phylotypes, herein comparisons relying in these attributes should be avoided across different preservation and preparation methodologies. Regardless of the methodology employed, the variability among coral colonies, as shown in the current study, raises the importance of adequate colony replication (Gray et al., 2013 and this study). Our results demonstrate the importance of replication when assessing the relative abundance and persistence of dominant or key bacteria. As has been explored in the literature 16S rRNA amplicon sequencing has, as any sampling method, caveats, and bias (Hamady and Knight, 2009), and focusing on one attribute of the community limits the overall picture of the bacterial community. The literature offers many alternatives that vary in the degree of importance to the abundance and occurrence of bacterial phylotypes; e.g., Abundance-Ubiquity test (Hester et al., 2015), core microbiome (Ainsworth et al., 2015; Hernandez-Agreda et al., 2017) and indicator species (De Cáceres and Legendre, 2009). If the purpose of the study is to identify key bacterial species, the use of 16S rRNA amplicon and the exploration of relative abundance as well as the occurrence of individual bacteria will contribute to select small groups of bacterial phylotypes over which hypotheses can be raised (e.g., out of 100,000s bacterial phylotypes, select a group 20–100 bacterial OTUs for further exploration). The determination of the importance of those bacteria and the characteristics of the potential symbiosis with the host will depend on further analysis of the niche occupation, metabolic, and physiological characteristics and the determination of a real symbiosis.

## Author Contributions

AH-A, WL, and TA: conceptual approach, design and manuscript writing. AH-A: collection, identification of coral individuals, data analysis, and interpretation.

## Conflict of Interest Statement

The authors declare that the research was conducted in the absence of any commercial or financial relationships that could be construed as a potential conflict of interest.

## Footnotes

1 http://bioinformatics.psb.ugent.be/webtools/Venn/
