# Supplementary material for: A Comparative Analysis of Microbial DNA Preparation Methods for Use With Massive and Branching Coral Growth Forms
Source: Front Microbiol. 2018 Sep 7;9:2146. doi: 10.3389/fmicb.2018.02146 (PMC6137167; doi:10.3389/fmicb.2018.02146)
Supplement: Supplementary file 1 [file Data_Sheet_1.pdf]

Supplementary Material

A comparative analysis of microbial DNA preparation methods for use with massive and branching coral growth forms

Alejandra Hernandez-Agreda[1,2#*], William Leggat[1,2,3], Tracy D. Ainsworth[1,4]

[1] Australian Research Council Centre of Excellence for Coral Reef Studies, James Cook University, Townsville, QLD, Australia, [2] The College of Public Health, Medical and Veterinary Sciences, James Cook University, Townsville, QLD, Australia, [3] School of Environmental and Life Sciences, The University of Newcastle, Ourimbah, NSW, Australia, [4] School of Biological, Earth and Environmental Sciences, University of New South Wales, Sydney, NSW, Australia.

* Correspondence:
Alejandra Hernandez-Agreda
alejandra.hernandezagreda@my.jcu.edu.au

[#] Current address: California Academy of Sciences, San Francisco, California, USA.

1.1 Supplementary Figures

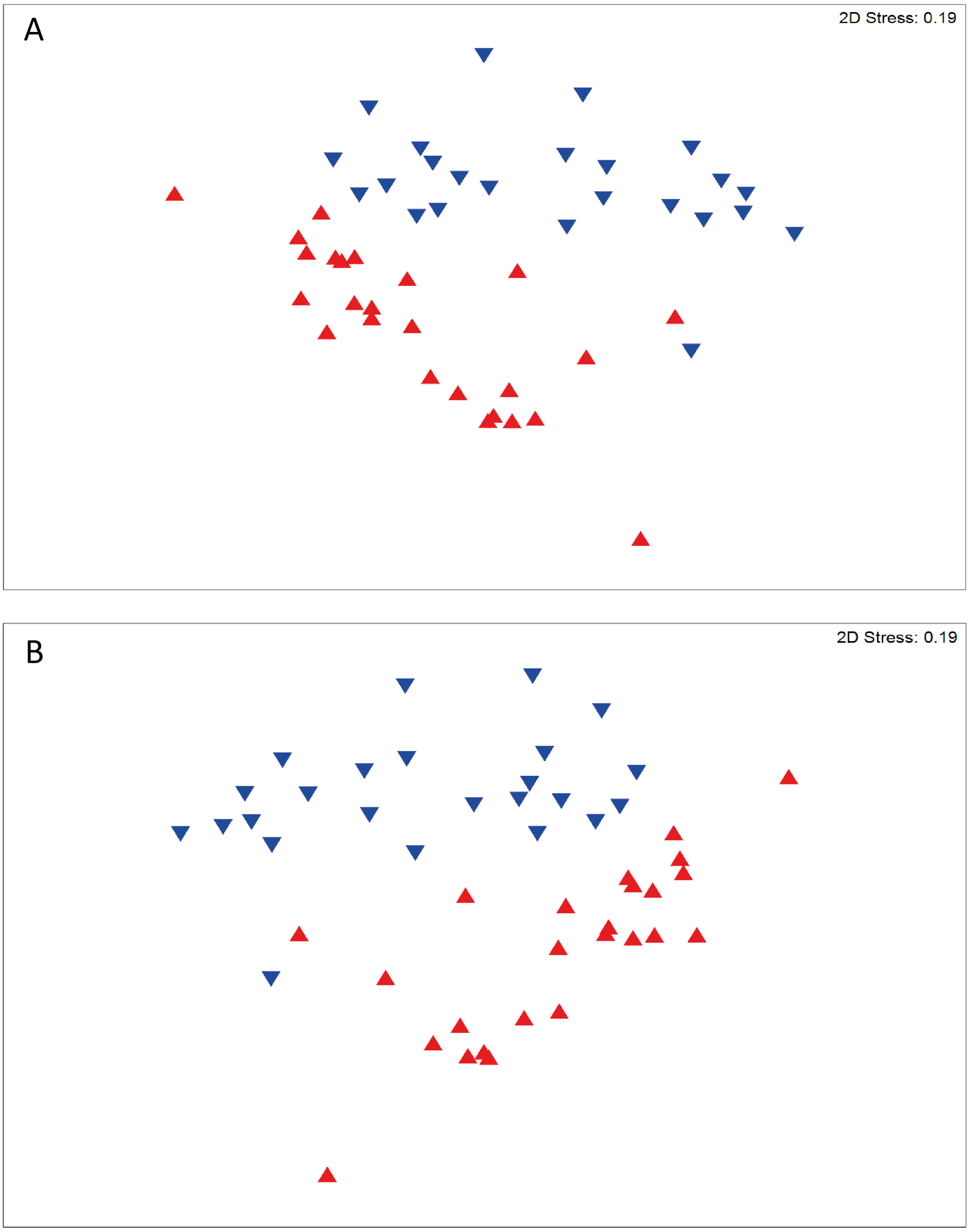

Supplementary Figure 1. Bacterial communities are different between coral species. Non-metric MDS based on relative abundance (A) and presence/absence data (B). nMDS are based on Bray-Curtis dissimilarity of fourth root transformed data (A) and Sorensen dissimilarity (B). Bacterial assemblage structure is different among coral species. Blue: *G. edwardsi*, red: *I. palifera*. Statistical analysis in Supp. Table 3, 4.

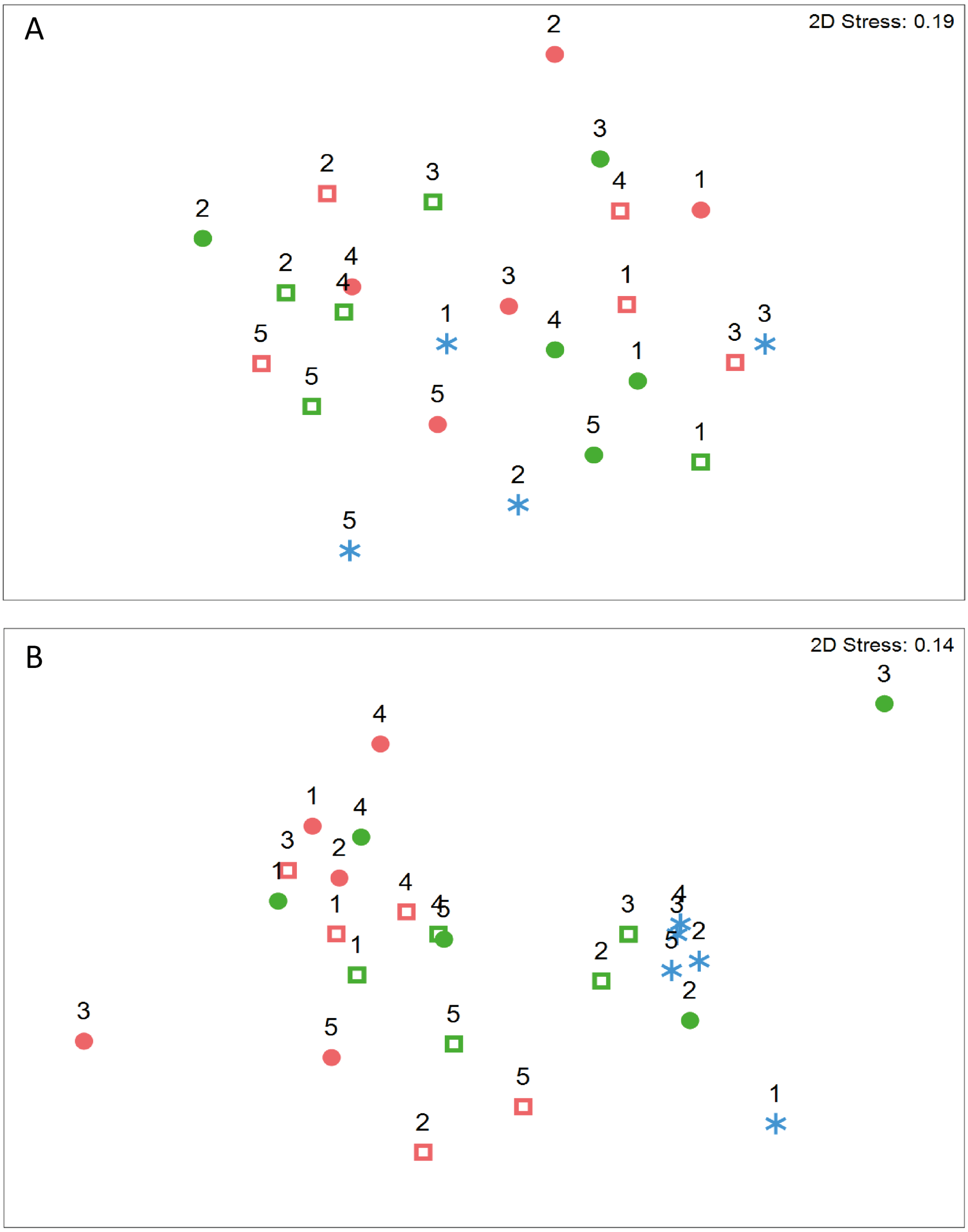

Supplementary Figure 2. Non-metric MDS based on presence/absence data for *G. edwardsi* (A) and *I. palifera* (B). No differences are observed in the composition of *G. edwardsi*. For *I. palifera*, there are differences in composition between fragments treated with PFA-decalcification (blue starts) and fragments preserved with DMSO, regardless the homogenization method. nMDS are based on Sorensen dissimilarity. Green: Liquid nitrogen, red: DMSO, blue: PFA, circles: bead beating, squares: crushing. Colonies indicated with numbers. Supporting analyses in Supp. Table 6, 8, 10, 12.

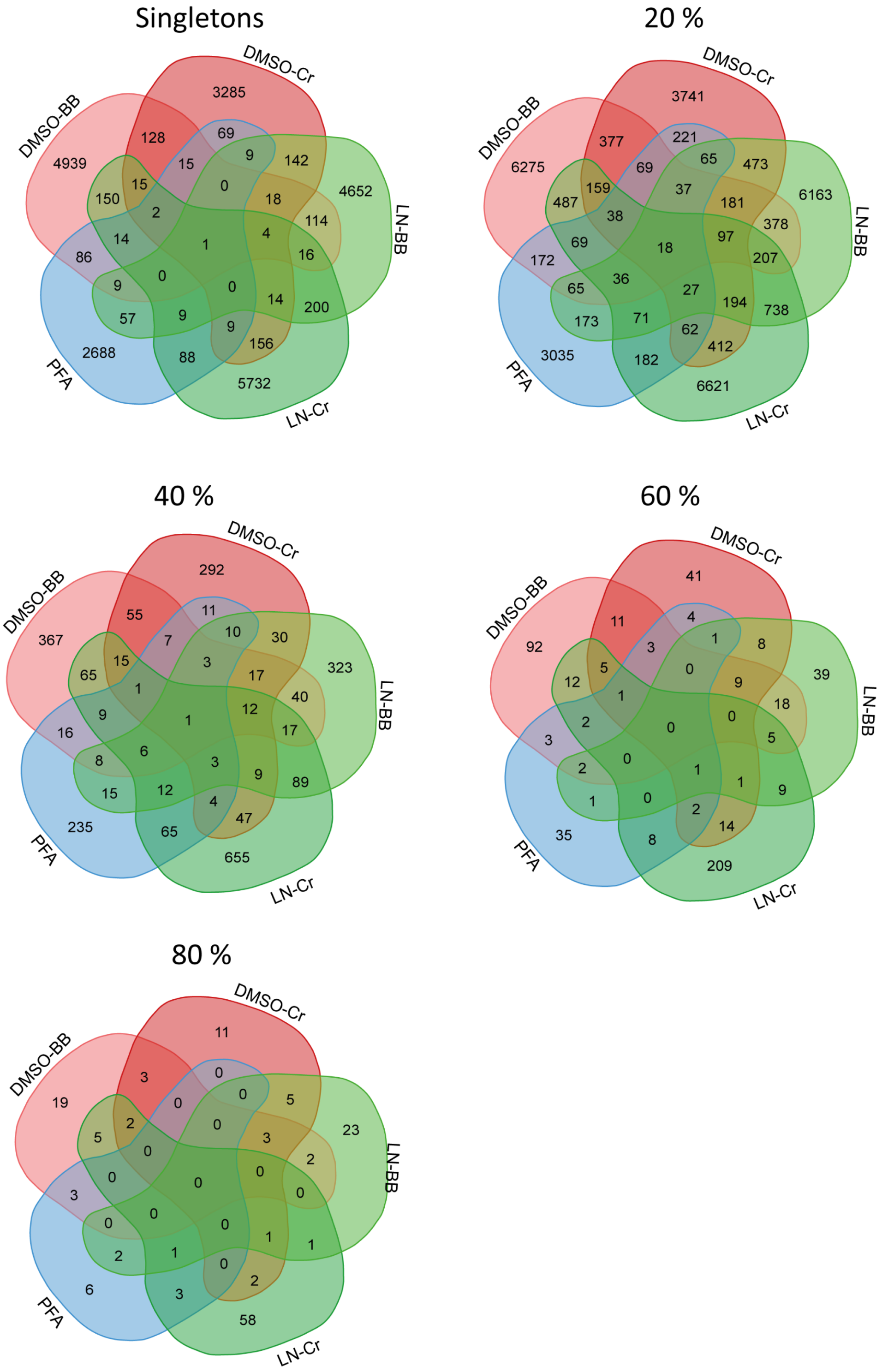

Supplementary Figure 3. Venn diagram for singletons and bacterial assemblages persistent at 20, 40, 60, 80% of the samples of each methodology considered for preservation and homogenization in *G. edwardsi*. Bacterial phylotypes analyzed at different percentages of persistence seems to show distinct bacterial assemblages, since the number of phylotypes detected by only one combination of preservation and homogenization method is superior to those shared between distinct methods. However, analyses of the structure of bacterial assemblages (Figure 3A) demonstrate that there are no differences and there are common phylotypes among methods, but

their persistence and relative abundance vary among preservation and homogenization treatments (Figure 4A, C, E, G, I).

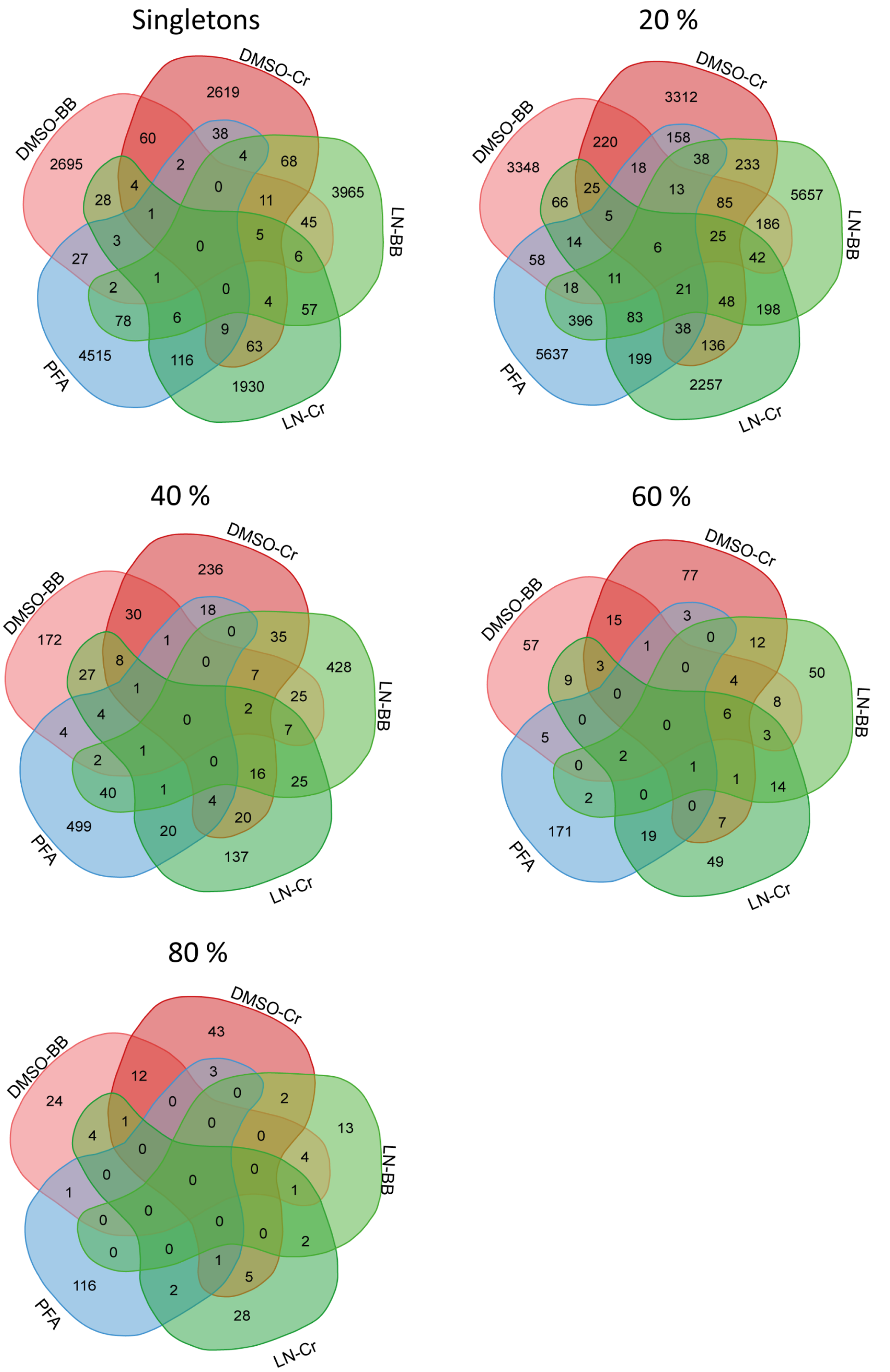

Supplementary Figure 4. Venn diagram for singletons and bacterial assemblages persistent at 20, 40, 60, 80% of the samples of each methodology considered for preservation and homogenization in *I. palifera*. As observed in *G. edwardsi* (previous image) analysis of bacterial phylotypes considering different percentages of persistence indicate distinct bacterial assemblages. However, community structure

analysis only detected differences between PFA-decalcified and both homogenization treatments preserved in DMSO (Figure 3B). All the preservation methods have phylotypes in common, but they are differentiated by low occurrence, low abundance phylotypes (Figure 4B, D, F, H, J).

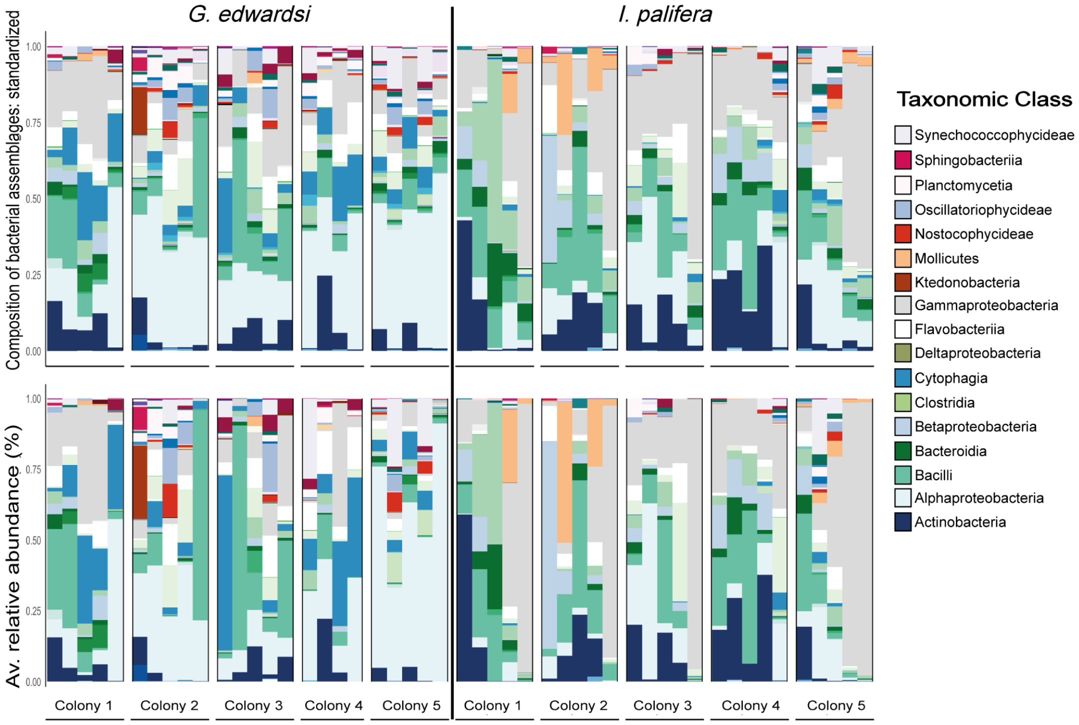

Supplementary Figure 5. Variation of taxonomic composition (top) and structure (bottom) among preservation and homogenization methods by colony. There is a high variability across colonies in taxonomic composition and structure. Analyzing each colony individually, the comparison among preservation and homogenization methods shows a consistent composition among them for *G. edwardsi* (top left) despite patterns in the structure (bottom left) are not. For *I. palifera*, patterns in taxonomic composition and structure differ among colonies, but colonies 3-5 show consistency in dominant classes among preservation and homogenization methods (top and bottom right). For each colony, columns are ordered as follows: DMSO-Bead beating, DMSO-crushing, LN-Bead beating, LN-crushing, PFA-decalcification. Major taxonomic classes are presented in the legend, for complete legend see Supp. Figure 6.

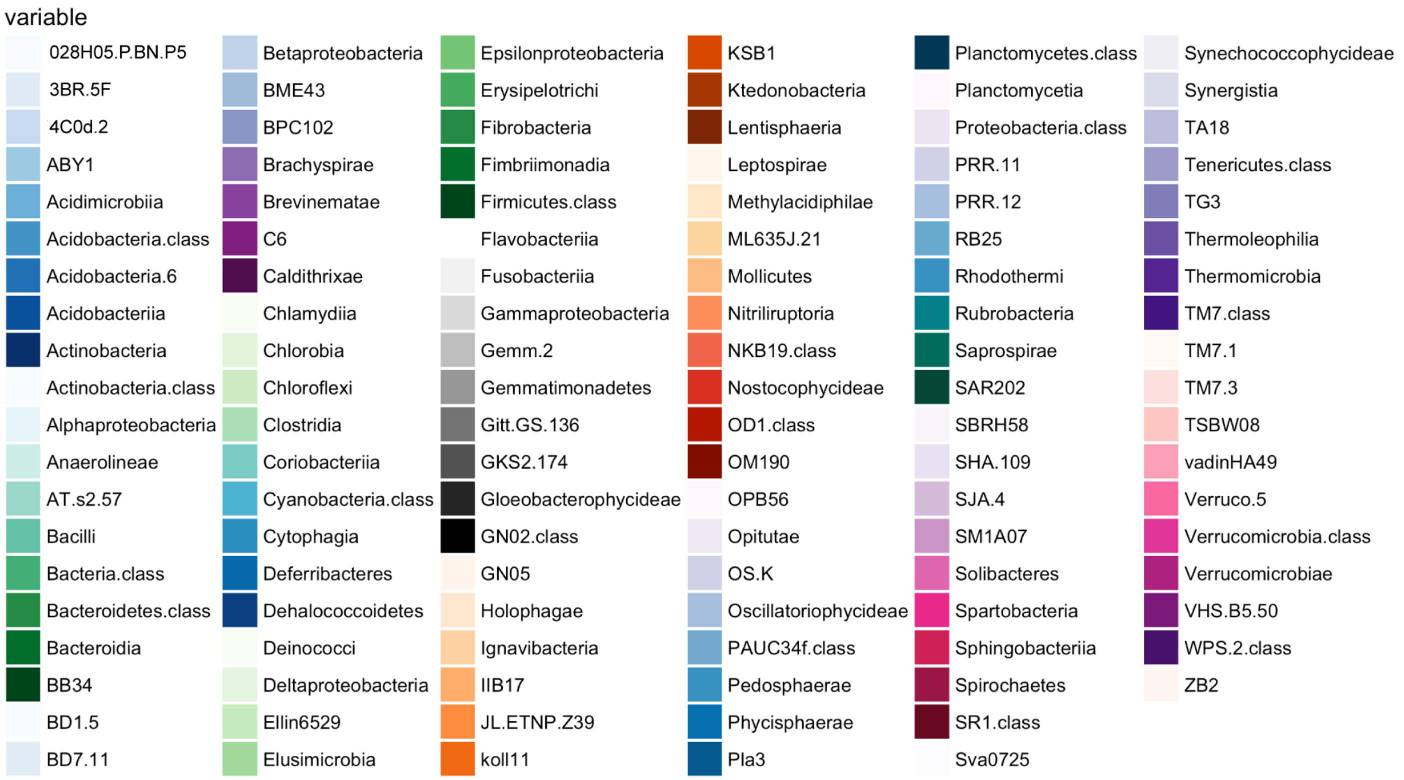

Supplementary Figure 6 – Legend of all taxonomic classes considered in Figure 6 and Supp. Figure 5.

1.2 Supplementary Tables

Supplementary Table 1. Permutational analysis of variance (univariate PERMANOVA) on diversity indices for *G. edwardsi* microbiome. The test is based on Euclidean distances, performed using 9,999 permutations to compare Preservation and Homogenization methods. A) Fully crossed design Preservation (Pr; DMSO and LN) x Homogenization (Ho; BB and Cr). B) Comparison between PFA versus the other preservation and homogenization methods. Bonferroni *p*-value for four comparisons 0.0125. P(perm): *P*-value based on permutations, U. perms: Unique permutations, P(MC): Monte Carlo *P*- value, ECV(%): Estimated components of variation.

Richness (d) - Margalef's index

Source	df	SS	MS	Pseudo-F	P(perm)	Unique perms	P(MC)	ECV(%)
Preservation (Pr)	1	7707.1	7707.1	0.36539	0.5844	9875	0.5526	0
Homogenization (Ho)	1	1.0604	1.0604	5.03E-05	0.9948	9856	0.9938	0
PrxHo	1	9845.5	9845.5	0.46677	0.5165	9813	0.5075	0
Residual	16	3.37E+05	21093					100
Total	19	3.55E+05						

PFA vs.	Source	df	SS	MS	Pseudo-F	P(perm)	Unique perms	P(MC)	ECV(%)
DMSO-BB	Treatment	1	11639	11639	3.379	0.0965	126	0.1088	42.3
	Residual	7	24112	3444.5					57.7
	Total	8	35750						
DMSO-Cr	Treatment	1	1799.5	1799.5	0.33739	0.5899	126	0.5819	0
	Residual	7	37335	5333.6					100
	Total	8	39135						
LN-BB	Treatment	1	10052	10052	0.34269	0.8945	126	0.5791	0
	Residual	7	2.05E+05	29334					100
	Total	8	2.15E+05						
LN-Cr	Treatment	1	27921	27921	1.816	0.2222	126	0.2122	30.0
	Residual	7	1.08E+05	15375					70.0
	Total	8	1.36E+05						

Diversity (H') - Shannon index

Source	df	SS	MS	Pseudo-F	P(perm)	Unique perms	P(MC)	ECV(%)
Preservation (Pr)	1	0.030301	0.030301	0.025459	0.8758	9866	0.8733	0.0
Homogenization (Ho)	1	1.3743	1.3743	1.1546	0.2971	9847	0.2998	11.1
PrxHo	1	0.35677	0.35677	0.29976	0.5862	9831	0.5929	0.0
Residual	16	19.043	1.1902					88.9
Total	19	20.805						

PFA vs.	Source	df	SS	MS	Pseudo-F	P(perm)	Unique perms	P(MC)	ECV(%)
DMSO-BB	Treatment	1	3.6559	3.6559	5.5533	0.0594	126	0.0498	50.3
	Residual	7	4.6083	0.65833					49.7
	Total	8	8.2642						
DMSO-Cr	Treatment	1	5.2687	5.2687	4.0689	0.0859	126	0.0838	45.4
	Residual	7	9.064	1.2949					54.6
	Total	8	14.333						
LN-BB	Treatment	1	1.9538	1.9538	1.66	0.2475	126	0.2428	27.8
	Residual	7	8.2391	1.177					72.2
	Total	8	10.193						
LN-Cr	Treatment	1	6.6436	6.6436	6.6373	0.0493	126	0.0363	53.0
	Residual	7	7.0067	1.001					47.0
	Total	8	13.65						

Evenness (J') - Pielou's evenness

Source	df	SS	MS	Pseudo-F	P(perm)	Unique perms	P(MC)	ECV(%)
Preservation (Pr)	1	3.73E-05	3.73E-05	0.0026071	0.9584	9852	0.9637	0.0
Homogenization (Ho)	1	0.031505	0.031505	2.205	0.1576	9843	0.1547	25.8
PrxHo	1	0.0019043	0.0019043	0.13327	0.7188	9840	0.7164	0.0
Residual	16	0.22861	0.014288					74.2
Total	19	0.26206						

PFA vs.	Source	df	SS	MS	Pseudo-F	P(perm)	Unique perms	P(MC)	ECV(%)
DMSO-BB	Treatment	1	0.038439	0.038439	2.5346	0.1488	126	0.1556	37.0
	Residual	7	0.10616	0.015165					63.0
	Total	8	0.1446						
DMSO-Cr	Treatment	1	0.081394	0.081394	3.7255	0.1017	126	0.0928	43.9
	Residual	7	0.15293	0.021848					56.1
	Total	8	0.23433						
LN-BB	Treatment	1	0.026535	0.026535	1.6316	0.2368	126	0.2375	27.4
	Residual	7	0.11384	0.016264					72.6
	Total	8	0.14038						
LN-Cr	Treatment	1	0.096299	0.096299	4.6292	0.0972	126	0.0635	47.5
	Residual	7	0.14562	0.020802					52.5
	Total	8	0.24192						

Av. Tax. Distinctness (Δ^+) - Average of taxonomic distinctness

Source	df	SS	MS	Pseudo-F	P(perm)	Unique perms	P(MC)	ECV(%)
Preservation (Pr)	1	8.97E-05	8.97E-05	6.40E-05	0.9927	9810	0.9929	0
Homogenization (Ho)	1	0.4404	0.4404	0.3146	0.5837	9838	0.5729	0
PrxHo	1	1.1805	1.1805	0.84326	0.3698	9832	0.3707	0
Residual	16	22.398	1.3999					100
Total	19	24.019						

PFA vs.	Source	df	SS	MS	Pseudo-F	P(perm)	Unique perms	P(MC)	ECV(%)
DMSO-BB	Treatment	1	1.7171	1.7171	3.3572	0.0907	126	0.1069	42.1
	Residual	7	3.5802	0.51146					57.9
	Total	8	5.2973						
DMSO-Cr	Treatment	1	0.020627	0.020627	0.012827	0.8747	126	0.9109	0
	Residual	7	11.257	1.6081					100
	Total	8	11.277						
LN-BB	Treatment	1	0.33608	0.33608	0.29218	0.5947	126	0.6027	0
	Residual	7	8.0519	1.1503					100
	Total	8	8.388						
LN-Cr	Treatment	1	0.74242	0.74242	0.39932	0.4819	126	0.5456	0
	Residual	7	13.015	1.8592					100
	Total	8	13.757						

Var. Tax. Distinctness (Λ^+) - Variation of taxonomic distinctness

Source	df	SS	MS	Pseudo-F	P(perm)	Unique perms	P(MC)	ECV(%)
Preservation (Pr)	1	241.96	241.96	0.48465	0.4909	9812	0.5047	0.0
Homogenization (Ho)	1	437.36	437.36	0.87606	0.3648	9829	0.3596	0.0
PrxHo	1	763.72	763.72	1.5298	0.2304	9823	0.2414	24.6
Residual	16	7987.7	499.23					75.4
Total	19	9430.8						

PFA vs.	Source	df	SS	MS	Pseudo-F	P(perm)	Unique perms	P(MC)	ECV(%)
DMSO-BB	Treatment	1	313.89	313.89	0.96318	0.3605	126	0.3598	0
	Residual	7	2281.2	325.89					100
	Total	8	2595.1						
DMSO-Cr	Treatment	1	214.59	214.59	0.33527	0.5874	126	0.5792	0
	Residual	7	4480.3	640.05					100
	Total	8	4694.9						
LN-BB	Treatment	1	93.378	93.378	0.22052	0.7485	126	0.6559	0
	Residual	7	2964.1	423.44					100
	Total	8	3057.5						
LN-Cr	Treatment	1	200.07	200.07	0.2184	0.6196	126	0.6591	0
	Residual	7	6412.6	916.08					100
	Total	8	6612.7						

Supplementary Table 2. Permutational analysis of variance (univariate PERMANOVA) on diversity indices for *I. palifera* microbiome. The test is based on Euclidean distances, performed using 9,999 permutations to compare Preservation and Homogenization methods. A) Fully crossed design Preservation (Pr; DMSO and LN) x Homogenization (Ho; BB and Cr). B) Comparison between PFA versus the other preservation and homogenization methods. Bonferroni *p*-value for four comparisons 0.0125. P(perm): *P*-value based on permutations, U. perms: Unique permutations, P(MC): Monte Carlo *P*- value, ECV(%): Estimated components of variation.

Richness (d) - Margalef's index

Source	df	SS	MS	Pseudo-F	P(perm)	Unique perms	P(MC)	ECV(%)
Preservation (Pr)	1	1185	1185	0.24861	0.7223	9898	0.6275	0.0
Homogenization (Ho)	1	4438.2	4438.2	0.93111	0.4012	9904	0.3549	0.0
PrxHo	1	9507.2	9507.2	1.9946	0.1735	9884	0.1807	30.8
Residual	16	76264	4766.5					69.2
Total	19	91395						

PFA vs.	Source	df	SS	MS	Pseudo-F	P(perm)	Unique perms	P(MC)	ECV(%)
DMSO-BB	Treatment	1	11593	11593	2.1292	0.2086	126	0.1821	32.2
	Residual	8	43559	5444.9					67.8
	Total	9	55152						
DMSO-Cr	Treatment	1	476.95	476.95	0.30134	0.7079	126	0.5941	0
	Residual	8	12662	1582.7					100
	Total	9	13139						
LN-BB	Treatment	1	206.91	206.91	0.017249	0.8089	126	0.8979	0
	Residual	8	95964	11995					100
	Total	9	96170						
LN-Cr	Treatment	1	17014	17014	3.907	0.0164	126	0.0816	43.3
	Residual	8	34838	4354.8					56.7
	Total	9	51852						

Diversity (H') - Shannon index

Source	df	SS	MS	Pseudo-F	P(perm)	Unique perms	P(MC)	ECV(%)
Preservation (Pr)	1	0.024867	0.024867	0.035762	0.8618	9826	0.8474	0.0
Homogenization (Ho)	1	0.45239	0.45239	0.6506	0.4253	9826	0.4281	0.0
PrxHo	1	2.5886	2.5886	3.7228	0.07	9850	0.0699	42.5
Residual	16	11.125	0.69534					57.5
Total	19	14.191						

PFA vs.	Source	df	SS	MS	Pseudo-F	P(perm)	Unique perms	P(MC)	ECV(%)
DMSO-BB	Treatment	1	4.4183	4.4183	3.1336	0.1802	126	0.1116	39.5
	Residual	8	11.28	1.41					60.5
	Total	9	15.698						
DMSO-Cr	Treatment	1	7.6399	7.6399	5.9756	0.0377	126	0.0407	49.9
	Residual	8	10.228	1.2785					50.1
	Total	9	17.868						
LN-BB	Treatment	1	9.7852	9.7852	8.4138	0.0407	126	0.0226	54.9
	Residual	8	9.304	1.163					45.1
	Total	9	19.089						
LN-Cr	Treatment	1	2.2948	2.2948	1.785	0.172	126	0.2162	28.4
	Residual	8	10.285	1.2856					71.6
	Total	9	12.58						

Evenness (J') - Pielou's evenness

Source	df	SS	MS	Pseudo-F	P(perm)	Unique perms	P(MC)	ECV(%)
Preservation (Pr)	1	0.0046901	0.0046901	0.31462	0.5915	9823	0.5837	0.0
Homogenization (Ho)	1	0.011814	0.011814	0.79247	0.389	9816	0.3959	0.0
PrxHo	1	0.015485	0.015485	1.0388	0.3435	9845	0.3264	8.1
Residual	16	0.23852	0.014907					91.9
Total	19	0.27051						

PFA vs.	Source	df	SS	MS	Pseudo-F	P(perm)	Unique perms	P(MC)	ECV(%)
DMSO-BB	Treatment	1	0.17517	0.17517	6.5829	0.0156	126	0.0344	51.4
	Residual	8	0.21287	0.026609					48.6
	Total	9	0.38804						
DMSO-Cr	Treatment	1	0.18461	0.18461	10.819	0.0312	126	0.0101	58.4
	Residual	8	0.13651	0.017064					41.6
	Total	9	0.32113						
LN-BB	Treatment	1	0.20985	0.20985	16.221	0.0202	126	0.0037	63.6
	Residual	8	0.1035	0.012937					36.4
	Total	9	0.31335						
LN-Cr	Treatment	1	0.085993	0.085993	4.0889	0.1044	126	0.0829	44.0
	Residual	8	0.16825	0.021031					56.0
	Total	9	0.25424						

Av. Tax. Distinctness (Δ^+) - Average of taxonomic distinctness

Source	df	SS	MS	Pseudo-F	P(perm)	Unique perms	P(MC)	ECV(%)
Preservation (Pr)	1	3.0033	3.0033	3.0136	0.1042	9842	0.1018	31.0
Homogenization (Ho)	1	0.6777	0.6777	0.68001	0.4194	9839	0.4228	0.0
PrxHo	1	0.20675	0.20675	0.20746	0.6444	9820	0.6543	0.0
Residual	16	15.946	0.9966					69.0
Total	19	19.833						

PFA vs.	Source	df	SS	MS	Pseudo-F	P(perm)	Unique perms	P(MC)	ECV(%)
DMSO-BB	Treatment	1	4.5375	4.5375	5.2746	0.0752	126	0.0524	48.0
	Residual	8	6.8821	0.86026					52.0
	Total	9	11.42						
DMSO-Cr	Treatment	1	5.7156	5.7156	13.68	0.0073	126	0.0072	61.4
	Residual	8	3.3424	0.4178					38.6
	Total	9	9.058						
LN-BB	Treatment	1	0.34012	0.34012	0.70347	0.4633	126	0.4263	0
	Residual	8	3.8679	0.48349					100
	Total	9	4.208						
LN-Cr	Treatment	1	2.2106	2.2106	3.1303	0.1101	125	0.111	39.5
	Residual	8	5.6496	0.7062					60.5
	Total	9	7.8602						

Var. Tax. Distinctness (Λ^+) - Variation of taxonomic distinctness

Source	df	SS	MS	Pseudo-F	P(perm)	Unique perms	P(MC)	ECV(%)
Preservation (Pr)	1	530.78	530.78	0.99775	0.3614	9856	0.3315	0
Homogenization (Ho)	1	329.4	329.4	0.61919	0.4686	9858	0.4407	0
PrxHo	1	0.29975	0.29975	0.00056347	0.9831	9854	0.9808	0
Residual	16	8511.6	531.98					100
Total	19	9372.1						

PFA vs.	Source	df	SS	MS	Pseudo-F	P(perm)	Unique perms	P(MC)	ECV(%)
DMSO-BB	Treatment	1	4435.6	4435.6	6.4742	0.0087	126	0.0347	51.1
	Residual	8	5481	685.13					48.9
	Total	9	9916.6						
DMSO-Cr	Treatment	1	2932.7	2932.7	67.943	0.0077	126	0.0001	78.5
	Residual	8	345.31	43.164					21.5
	Total	9	3278						
LN-BB	Treatment	1	2570.2	2570.2	13.759	0.0071	126	0.0059	61.5
	Residual	8	1494.3	186.79					38.5
	Total	9	4064.5						
LN-Cr	Treatment	1	1404.5	1404.5	6.6064	0.0489	126	0.0358	51.4
	Residual	8	1700.7	212.59					48.6
	Total	9	3105.2						

Supplementary Table 3. Permutational multivariate analysis of variance (PERMANOVA) for the relative abundance data based on Bray-Curtis dissimilarities. Test performed using 9,999 permutations. P(perm): *P*-value based on permutations, U. perms: Unique permutations, P(MC): Monte Carlo *P*- value, ECV(%): Estimated components of variation.

Source	df	SS	MS	Pseudo-F	P(perm)	Unique perms	P(MC)	ECV(%)
Preservation (Pr)	1	5179.7	5179.7	1.1766	0.2514	3	0.3333	6.19
Homogenization (Ho)	1	4083	4083	1.0305	0.2595	3	0.4714	2.44
Coral species (Co)	1	11077	11077	2.6999	0.0001	9834	0.0004	18.53
PrxHo	1	3991.9	3991.9	1.0396	0.2713	18	0.4726	3.87
PrxCo	1	4402.2	4402.2	1.073	0.2873	9814	0.3615	5.43
HoxCo	1	3962.1	3962.1	0.96571	0.5755	9792	0.5208	0.00
PrxHoxCo	1	3840	3840	0.93594	0.6585	9813	0.5547	0.00
Residual	32	1.31E+05	4102.8					63.55
Total	39	1.68E+05						

Supplementary Table 4. Permutational multivariate analysis of variance (PERMANOVA) for the compositional (Presence/Absence) data based on Sorensen dissimilarities. Test performed using 9,999 permutations. P(perm): *P*-value based on permutations, U. perms: Unique permutations, P(MC): Monte Carlo *P*- value, ECV(%): Estimated components of variation.

Source	df	SS	MS	Pseudo-F	P(perm)	Unique perms	P(MC)	ECV(%)
Preservation (Pr)	1	5005.1	5005.1	1.1692	0.2432	3	0.3295	6.16
Homogenization (Ho)	1	3964.9	3964.9	1.007	0.251	3	0.488	1.21
Coral species (Co)	1	10694	10694	2.6041	0.0001	9780	0.0003	18.59
PrxHo	1	3916.8	3916.8	1.0434	0.2427	18	0.4538	4.13
PrxCo	1	4280.9	4280.9	1.0424	0.3601	9808	0.402	4.27
HoxCo	1	3937.2	3937.2	0.9587	0.606	9797	0.533	0.00
PrxHoxCo	1	3753.9	3753.9	0.91408	0.7349	9810	0.5949	0.00
Residual	32	1.31E+05	4106.8					65.63
Total	39	1.67E+05						

Supplementary Table 5. Permutational multivariate analysis of variance (PERMANOVA) for the relative abundance of bacterial community associated with *G. edwardsi.* Analysis based on Bray-Curtis dissimilarities, excluding PFA-PBS treated samples. Test performed using 9,999 permutations. P(perm): *P*-value based on permutations, U. perms: Unique permutations, P(MC): Monte Carlo *P*- value, ECV(%): Estimated components of variation.

Source	df	SS	MS	Pseudo-F	P(perm)	Unique perms	P(MC)	ECV(%)
Preservation (Pr)	1	4514.2	4514.2	1.0527	0.3371	9843	0.4014	6.77
Homogenization (Ho)	1	3602.4	3602.4	0.84007	0.8329	9823	0.6192	0.00
PrxHo	1	4025.6	4025.6	0.93876	0.6294	9830	0.514	0.00
Residual	16	68611	4288.2					93.23
Total	19	80753						

Supplementary Table 6. Permutational multivariate analysis of variance (PERMANOVA) for the composition (Presence/Absence) of bacterial community associated with *G. edwardsi*. Analysis based on Sorensen dissimilarities, excluding PFA-PBS treated samples. Test performed using 9,999 permutations. P(perm): *P*-value based on permutations, U. perms: Unique permutations, P(MC): Monte Carlo *P*- value, ECV(%): Estimated components of variation.

Source	df	SS	MS	Pseudo-F	P(perm)	Unique perms	P(MC)	ECV(%)
Preservation (Pr)	1	4485	4485	1.053	0.3481	9827	0.397	6.79
Homogenization (Ho)	1	3544	3544	0.83206	0.8531	9820	0.6415	0.00
PrxHo	1	3836.6	3836.6	0.90077	0.7292	9830	0.5557	0.00
Residual	16	68148	4259.3					93.21
Total	19	80014						

Supplementary Table 7. Permutational multivariate analysis of variance (PERMANOVA) for the relative abundance to compare bacterial community associated with *G. edwardsi* preserved with PFA versus the other preservation and homogenization methods. Analysis based on Bray-Curtis dissimilarities. Test performed using 9,999 permutations. Bonferroni *p*-value for four comparisons 0.0125. P(perm): *P*-value based on permutations, U. perms: Unique permutations, P(MC): Monte Carlo *P*- value, ECV(%): Estimated components of variation.

PFA vs.	Source	df	SS	MS	Pseudo-F	P(perm)	Unique perms	P(MC)	ECV(%)
DMSO-BB	Treatment	1	4648.6	4648.6	1.0934	0.2496	126	0.3881	12.66
	Residual	7	29760	4251.4					87.34
	Total	8	34408						
DMSO-Cr	Treatment	1	4316.3	4316.3	1.0148	0.4094	126	0.4323	5.46
	Residual	7	29774	4253.4					94.54
	Total	8	34090						
LN-BB	Treatment	1	4949.4	4949.4	1.1885	0.1176	126	0.3179	17.08
	Residual	7	29152	4164.5					82.92
	Total	8	34101						
LN-Cr	Treatment	1	4914.3	4914.3	1.1738	0.0971	126	0.3243	16.51
	Residual	7	29308	4186.8					83.49
	Total	8	34222						

Supplementary Table 8. Permutational multivariate analysis of variance (PERMANOVA) for the composition (Presence/Absence) to compare bacterial community associated with *G. edwardsi* preserved with PFA versus the other preservation and homogenization methods. Analysis based on Sorensen dissimilarities. Test performed using 9,999 permutations. Bonferroni *p*-value for four comparisons 0.0125. P(perm): *P*-value based on permutations, U. perms: Unique permutations, P(MC): Monte Carlo *P*- value, ECV(%): Estimated components of variation.

PFA vs.	Source	df	SS	MS	Pseudo-F	P(perm)	Unique perms	P(MC)	ECV(%)
DMSO-BB	Treatment	1	4588.5	4588.5	1.0794	0.2446	126	0.3878	11.79
	Residual	7	29757	4251					88.21
	Total	8	34346						
DMSO-Cr	Treatment	1	4256.1	4256.1	0.9979	0.4246	126	0.4444	0
	Residual	7	29855	4265.1					100
	Total	8	34112						
LN-BB	Treatment	1	4688.5	4688.5	1.1157	0.2062	126	0.3649	13.89
	Residual	7	29417	4202.4					86.11
	Total	8	34105						
LN-Cr	Treatment	1	4741.6	4741.6	1.1336	0.1116	126	0.3536	14.78
	Residual	7	29280	4182.8					85.22
	Total	8	34021						

Supplementary Table 9. Permutational multivariate analysis of variance (PERMANOVA) for the relative abundance of bacterial community associated with *I. palifera.* Analysis based on Bray-Curtis dissimilarities, excluding PFA-PBS treated samples. Test performed using 9,999 permutations. P(perm): *P*-value based on permutations, U. perms: Unique permutations, P(MC): Monte Carlo *P*- value, ECV(%): Estimated components of variation.

Source	df	SS	MS	Pseudo-F	P(perm)	Unique perms	P(MC)	ECV(%)
Preservation (Pr)	1	5067.8	5067.8	1.2937	0.0557	9832	0.2036	13.31
Homogenization (Ho)	1	4442.7	4442.7	1.1341	0.1959	9834	0.3224	9.00
PrxHo	1	3806.3	3806.3	0.97164	0.5716	9836	0.4778	0.00
Residual	16	62678	3917.4					77.69
Total	19	75995						

Supplementary Table 10. Permutational multivariate analysis of variance (PERMANOVA) for the composition (Presence/Absence) of bacterial community associated with *I. palifera* Analysis based on Sorensen dissimilarities, excluding PFA-PBS treated samples. Test performed using 9,999 permutations. P(perm): *P*-value based on permutations, U. perms: Unique permutations, P(MC): Monte Carlo *P*- value, ECV(%): Estimated components of variation.

Source	df	SS	MS	Pseudo-F	P(perm)	Unique perms	P(MC)	ECV(%)
Preservation (Pr)	1	4801	4801	1.2141	0.0948	9822	0.2571	11.73
Homogenization (Ho)	1	4358.1	4358.1	1.1021	0.2402	9815	0.354	8.10
PrxHo	1	3834.1	3834.1	0.96962	0.602	9817	0.4855	0.00
Residual	16	63268	3954.3					80.17
Total	19	76261						

Supplementary Table 11. Permutational multivariate analysis of variance (PERMANOVA) for the relative abundance to compare bacterial community associated with *I. palifera* preserved with PFA versus the other preservation and homogenization methods. Analysis based on Bray-Curtis dissimilarities. Test performed using 9,999 permutations. Bonferroni *p*-value for four comparisons 0.0125. P(perm): *P*-value based on permutations, U. perms: Unique permutations, P(MC): Monte Carlo *P*- value, ECV(%): Estimated components of variation.

PFA vs.	Source	df	SS	MS	Pseudo-F	P(perm)	Unique perms	P(MC)	ECV(%)
DMSO-BB	Treatment	1	8919.8	8919.8	2.5166	0.0084	126	0.0259	35.51
	Residual	8	28355	3544.4					64.49
	Total	9	37275						
DMSO-Cr	Treatment	1	8187.3	8187.3	2.3589	0.008	126	0.0339	34.27
	Residual	8	27767	3470.9					65.73
	Total	9	35954						
LN-BB	Treatment	1	6417.9	6417.9	1.7631	0.014	126	0.1096	28.09
	Residual	8	29121	3640.1					71.91
	Total	9	35539						
LN-Cr	Treatment	1	5040.4	5040.4	1.5268	0.0299	126	0.1642	24.50
	Residual	8	26411	3301.4					75.50
	Total	9	31452						

Supplementary Table 12. Permutational multivariate analysis of variance (PERMANOVA) for the composition (Presence/Absence) to compare bacterial community associated with *I. palifera* preserved with PFA versus the other preservation and homogenization methods. Analysis based on Sorensen dissimilarities. Test performed using 9,999 permutations. Bonferroni *p*-value for four comparisons 0.0125. P(perm): *P*-value based on permutations, U. perms: Unique permutations, P(MC): Monte Carlo *P*- value, ECV(%): Estimated components of variation.

**PFA vs.**	**Source**	**df**	**SS**	**MS**	**Pseudo-F**	**P(perm)**	**Unique perms**	**P(MC)**	**ECV(%)**
DMSO-BB	Treatment	1	8113.1	8113.1	2.1897	0.0068	126	0.043	32.79
	Residual	8	29640	3705					67.21
	Total	9	37753						
DMSO-Cr	Treatment	1	7580.3	7580.3	2.1124	0.0076	126	0.0481	32.05
	Residual	8	28707	3588.4					67.95
	Total	9	36287						
LN-BB	Treatment	1	6073.8	6073.8	1.6129	0.0181	126	0.1376	25.93
	Residual	8	30126	3765.7					74.07
	Total	9	36200						
LN-Cr	Treatment	1	5247.1	5247.1	1.5081	0.0271	126	0.1671	24.17
	Residual	8	27834	3479.2					75.83
	Total	9	33081						

Supplementary Table 13. Number of bacterial phylotypes per percentage of occurrence. *For. *G. edwardsi* in the treatment PFA-decalcified *n*=4, thus percentages of occurrence are 25%, 50%, 75%, 100%. OTUs: Operational Taxonomic Units.

**Coral species**	**Method**		**Singleton OTUs**	**Number of OTUs per percentage of occurrence**					**Total OTUs**
	**Preservation**	**Homogenization**		**20%**	**40%**	**60%**	**80%**	**100%**	
G. edwardsi	DMSO	Bead beating	5,511	8,665	639	163	37	18	9,522
	DMSO	Crushing	3,867	6,171	517	101	27	7	6,823
	Liquid nitrogen	Bead beating	5,245	8,923	595	94	38	8	9,658
	Liquid nitrogen	Crushing	6,410	9,418	1,010	269	73	20	10,790
	PFA	Decalcified	3,056	4,340*	406*	63*	15*		4,824
I. palifera	DMSO	Bead beating	2,890	4,140	291	113	47	21	4,612
	DMSO	Crushing	2,888	4,381	378	130	67	23	4,979
	Liquid nitrogen	Bead beating	4,252	7,060	589	103	22	13	7,787
	Liquid nitrogen	Crushing	2,233	3,174	273	114	44	42	3,647
	PFA	Decalcified	4,802	6,713	595	204	123	134	7,769

Supp. Table 14. Taxonomic identification of OTUs part of the Core 100% (A), dominant phylotypes (relative abundance ≥ 0.05, B) and top 10 dominant phylotypes (C) in *G. edwardsi* bacterial assemblage. OTU: Operational Taxonomic Units.

A) Core 100% - *G. edwardsi*

**Taxa**	**DMSO-BB**	**DMSO-Cr**	**LL-BB**	**LL-Cr**	**PFA**
Anoxybacillus kestanbolensis					OTU_1211, OTU_1300, OTU_2851
Bacteroides uniformis				OTU_2317	
Class Alphaproteobacteria	OTU_44776, OTU_44920				OTU_33247
Family Aerococcaceae	OTU_1854				
Family Endozoicimonaceae	OTU_207	OTU_207	OTU_54, OTU_207	OTU_54, OTU_1924	OTU_207
Family Phyllobacteriaceae		OTU_284			OTU_56231
Family Rhodobacteraceae					OTU_6265
Family Ruminococcaceae					OTU_757
Family Spirochaetaceae					OTU_7124
Genus *Bacteroides*				OTU_9499	
Genus *Diaphorobacter*	OTU_3474	OTU_3474		OTU_3474	OTU_3474
Genus *Erythrobacter*	OTU_769			OTU_769	
Genus *Halomicronema*	OTU_169, OTU_748, OTU_21418				
Genus *Marinomonas*	OTU_946				
Genus *Muricauda*	OTU_957				
Genus *Ruegeria*					OTU_63604
Genus SGUS912		OTU_73, OTU_6055	OTU_73	OTU_73, OTU_896, OTU_6009, OTU_6055, OTU_6132, OTU_15786, OTU_15792, OTU_15979, OTU_16008	
Order Gemellales	OTU_6137				
Order Kiloniellales	OTU_256, OTU_44796		OTU_256		OTU_256
Propionibacterium acnes	OTU_5472, OTU_29486, OTU_33911, OTU_34038	OTU_5472, OTU_32607	OTU_5472, OTU_34191	OTU_5472, OTU_29486, OTU_33913	OTU_5472
Pseudomonas veronii			OTU_7093, OTU_19203	OTU_7093	OTU_7093
Staphylococcus epidermidis					OTU_2781
Stenotrophomonas geniculata				OTU_5826	

B) Dominant phylotypes - *G. edwardsi*

Taxa	DMSO-BB	DMSO-Cr	LL-BB	LL-Cr	PFA
Acinetobacter johnsonii	OTU_15208				
Acinetobacter lwoffii		OTU_5687, OTU_12395			OTU_5687
Anoxybacillus kestanbolensis		OTU_1211, OTU_1223, OTU_1300, OTU_2563, OTU_2842, OTU_2843, OTU_2846, OTU_2847, OTU_2851, OTU_2859, OTU_2913, OTU_2915, OTU_4436			OTU_1211, OTU_1223, OTU_1300, OTU_2563, OTU_2842, OTU_2843, OTU_2845, OTU_2846, OTU_2847, OTU_2848, OTU_2850, OTU_2851, OTU_2856, OTU_2859, OTU_2909, OTU_2910, OTU_2913, OTU_2914, OTU_2915, OTU_2916, OTU_3522, OTU_3583, OTU_3584, OTU_3593, OTU_3958, OTU_3966, OTU_3983, OTU_3991, OTU_4003, OTU_4004, OTU_4012, OTU_4030, OTU_4042, OTU_4064, OTU_4077, OTU_4088, OTU_4093, OTU_4095, OTU_4108, OTU_4119, OTU_4135, OTU_4141, OTU_4166, OTU_4170, OTU_4182, OTU_4186, OTU_4203, OTU_4204, OTU_4205, OTU_4207, OTU_4213, OTU_4214, OTU_4216, OTU_4238, OTU_4240, OTU_4243, OTU_4244, OTU_4422, OTU_4427, OTU_4430, OTU_4431, OTU_4436, OTU_4452, OTU_4455, OTU_4511, OTU_5246, OTU_5643, OTU_7321
Ascidianibacter aurantiacus		OTU_6506		OTU_6506, OTU_7050	
Bacillus cereus		OTU_2673			
Bacillus thermoamylovorans	OTU_2883				
Bacteroides ovatus		OTU_6383			OTU_6383

Taxa	DMSO-BB	DMSO-Cr	LL-BB	LL-Cr	PFA
Bacteroides uniformis			OTU_2317	OTU_2317	OTU_2317
Bifidobacterium pseudolongum			OTU_15681		
Brevibacterium aureum			OTU_34884		
Brevundimonas diminuta		OTU_23567			OTU_23567
Class Alphaproteobacteria	OTU_43157	OTU_43157, OTU_44776, OTU_44920, OTU_46992	OTU_29134, OTU_33653, OTU_44920, OTU_50122	OTU_33247, OTU_33653, OTU_34012, OTU_34292, OTU_52955, OTU_71948	OTU_29134, OTU_33247, OTU_34886, OTU_40855, OTU_43157, OTU_43513, OTU_43789, OTU_43801, OTU_44776, OTU_50121, OTU_56285, OTU_64829, OTU_69119
Class Gammaproteobacteria			OTU_13285		
Class ML635J-21		OTU_46929			
Class Mollicutes			OTU_37		
Class SJA-4			OTU_23110		
Clostridium perfringens			OTU_39062		
Coccinimonas marina	OTU_9053		OTU_9053	OTU_9053	
Coralibacter albidoflavus			OTU_63		
Desulfovibrio capillatus		OTU_1207		OTU_604, OTU_1207	
Endozoicomonas montiporae					OTU_1494
Enterovibrio coralii					OTU_2229
Eubacterium dolichum		OTU_2899			
Family A4b	OTU_10191				
Family Aerococcaceae	OTU_1854, OTU_2944	OTU_10, OTU_786, OTU_1854, OTU_2720, OTU_2891, OTU_2944, OTU_3060	OTU_10, OTU_786, OTU_1854, OTU_2369, OTU_2718, OTU_2720, OTU_2750, OTU_2944, OTU_3559, OTU_3659	OTU_786, OTU_1854, OTU_2720, OTU_2944, OTU_3060, OTU_4643	OTU_2944
Family Alteromonadaceae			OTU_32037		
Family Anaplasmataceae				OTU_40563	
Family Bacillaceae					OTU_29137
Family Bacteriovoracaceae			OTU_2388		
Family Beijerinckiaceae		OTU_22295			
Family Chromatiaceae				OTU_31368	
Family Cohaesibacteraceae		OTU_43697, OTU_46932			OTU_899
Family Coxiellaceae			OTU_7718		
Family Desulfobulbaceae		OTU_6087		OTU_6087	
Family Desulfovibrionaceae			OTU_3832		OTU_16242, OTU_21775
Family Endozoicimonaceae	OTU_54, OTU_207	OTU_54, OTU_207	OTU_54, OTU_157, OTU_207, OTU_208, OTU_209, OTU_210, OTU_211, OTU_212,	OTU_54, OTU_157, OTU_207, OTU_209, OTU_210, OTU_211, OTU_212, OTU_229,	OTU_207, OTU_1793, OTU_3485, OTU_7268, OTU_7561

Taxa	DMSO-BB	DMSO-Cr	LL-BB	LL-Cr	PFA
			OTU_215, OTU_226, OTU_229, OTU_230, OTU_255, OTU_277, OTU_297, OTU_300, OTU_333, OTU_334, OTU_338, OTU_344, OTU_348, OTU_363, OTU_375, OTU_398, OTU_409, OTU_432, OTU_437, OTU_441, OTU_456, OTU_459, OTU_460, OTU_478, OTU_483, OTU_492, OTU_505, OTU_577, OTU_579, OTU_597, OTU_598, OTU_612, OTU_1587, OTU_1730, OTU_1775, OTU_1793, OTU_1924, OTU_7561, OTU_7735	OTU_230, OTU_255, OTU_297, OTU_300, OTU_333, OTU_334, OTU_338, OTU_344, OTU_348, OTU_363, OTU_375, OTU_398, OTU_422, OTU_432, OTU_437, OTU_441, OTU_456, OTU_459, OTU_474, OTU_483, OTU_490, OTU_492, OTU_577, OTU_597, OTU_598, OTU_612, OTU_1730, OTU_1793, OTU_1924, OTU_1926, OTU_6488, OTU_6989, OTU_7268, OTU_7561, OTU_9073, OTU_9124, OTU_12314, OTU_12701, OTU_12702	
Family Enterobacteriaceae			OTU_6441		
Family Flammeovirgaceae	OTU_17, OTU_5773, OTU_19149, OTU_22521, OTU_25155, OTU_26892, OTU_27759, OTU_28368	OTU_17, OTU_5773, OTU_7264, OTU_16509, OTU_22521	OTU_17, OTU_5773, OTU_22521, OTU_23170	OTU_17, OTU_15950, OTU_27543	OTU_17, OTU_5773, OTU_7264, OTU_14964, OTU_21595, OTU_22521, OTU_26892, OTU_27759, OTU_28148, OTU_28149
Family Flavobacteriaceae		OTU_166, OTU_23510	OTU_166, OTU_778, OTU_6266, OTU_19177	OTU_778, OTU_7593, OTU_11206, OTU_11573, OTU_16725, OTU_27531, OTU_30712, OTU_30720, OTU_30722, OTU_30743	OTU_5821, OTU_16774
Family Halomonadaceae				OTU_2294	
Family Helicobacteraceae					OTU_33792
Family Hyphomicrobiaceae	OTU_33305	OTU_33305	OTU_47287	OTU_47287	OTU_63617, OTU_69340
Family Ktedonobacteraceae	OTU_31667				
Family Lachnospiraceae		OTU_2043			
Family Lentisphaeraceae			OTU_45330		OTU_43097
Family Methylobacteriaceae		OTU_15477			
Family Neisseriaceae				OTU_6489	
Family Peptostreptococcaceae					OTU_45883, OTU_47374
Family Phyllobacteriaceae	OTU_284, OTU_33251	OTU_284, OTU_33251, OTU_71604	OTU_284, OTU_33251, OTU_34933, OTU_49682	OTU_284, OTU_26957, OTU_33251	OTU_284, OTU_17868, OTU_43816, OTU_56231, OTU_63620, OTU_66109

Taxa	DMSO-BB	DMSO-Cr	LL-BB	LL-Cr	PFA
Family Pirellulaceae		OTU_19684, OTU_26983			
Family Piscirickettsiaceae			OTU_2331, OTU_13280	OTU_2331	
Family Porphyromonadaceae	OTU_6666				
Family Propionibacteriaceae	OTU_22509				
Family Pseudanabaenaceae	OTU_58847			OTU_24704	
Family Pseudoalteromonadaceae			OTU_2193		OTU_2193, OTU_3100, OTU_3219
Family Pseudomonadaceae					OTU_2900, OTU_6533
Family Rhodobacteraceae	OTU_6265, OTU_46927, OTU_63155, OTU_66625	OTU_6265, OTU_31981, OTU_33726, OTU_43572, OTU_53182, OTU_59408	OTU_6265, OTU_25157, OTU_31981, OTU_43777, OTU_46927, OTU_67656, OTU_68288, OTU_73060, OTU_43300	OTU_6265, OTU_8247, OTU_25157, OTU_47292, OTU_47497, OTU_65604, OTU_72843	OTU_6265, OTU_46927, OTU_59408, OTU_65604, OTU_68288
Family Rhodospirillaceae			OTU_43300		OTU_48923, OTU_49577
Family Rikenellaceae			OTU_2323	OTU_2323	
Family Ruminococcaceae		OTU_5732			OTU_757, OTU_5732
Family Spirochaetaceae	OTU_1269, OTU_3183, OTU_7124	OTU_1269		OTU_1269, OTU_3183, OTU_8938	OTU_1269, OTU_3183, OTU_7124, OTU_7263, OTU_10594, OTU_11472, OTU_11931, OTU_12162
Family Vibrionaceae					OTU_2872, OTU_3211, OTU_6066, OTU_7269
Family Weeksellaceae		OTU_10762, OTU_15533			
Family Xenococcaceae			OTU_885, OTU_44423, OTU_44455, OTU_46980	OTU_885, OTU_44423, OTU_44455	
Genus 02d06			OTU_39190		OTU_39282
Genus *Acinetobacter*	OTU_12387				OTU_6019
Genus *Actinomyces*				OTU_16723	
Genus *Alcanivorax*					OTU_7429
Genus *Anabaena*			OTU_63517		
Genus *Anaerococcus*			OTU_23868, OTU_28565		
Genus *Anaerospora*		OTU_32870	OTU_32870		OTU_32870
Genus *Aquimarina*			OTU_9048	OTU_9048	
Genus *Bacillus*					OTU_3193, OTU_4174, OTU_5133, OTU_5145
Genus *Bacteroides*		OTU_6168, OTU_9499		OTU_9499	
Genus Candidatus Portiera				OTU_18933	
Genus *Capnocytophaga*				OTU_11205, OTU_27542	
Genus *Chryseobacterium*	OTU_25925				
Genus *Cloacibacterium*		OTU_970, OTU_22145	OTU_22145		OTU_970, OTU_1513, OTU_10346, OTU_23664

**Taxa**	**DMSO-BB**	**DMSO-Cr**	**LL-BB**	**LL-Cr**	**PFA**
Genus *Clostridium*			OTU_12675, OTU_38759, OTU_39191		
Genus *Comamonas*				OTU_16055, OTU_19845, OTU_21444	
Genus *Congregibacter*			OTU_19301	OTU_6508, OTU_21388	OTU_19830, OTU_21388
Genus *Coprococcus*				OTU_30745	
Genus *Corynebacterium*	OTU_6790, OTU_33744, OTU_36716	OTU_6790, OTU_33744, OTU_34477	OTU_12410, OTU_30862, OTU_37089	OTU_12410, OTU_16057, OTU_30862	OTU_6790, OTU_12410, OTU_17918, OTU_33744, OTU_6790
Genus *Delftia*			OTU_18355	OTU_18355, OTU_25878	
Genus *Diaphorobacter*	OTU_3474	OTU_969, OTU_3474, OTU_18563, OTU_19565	OTU_3474, OTU_18563	OTU_3474	OTU_3474
Genus *Enhydrobacter*		OTU_23505			
Genus *Erythrobacter*	OTU_769	OTU_769	OTU_769, OTU_63345	OTU_769	
Genus *Exiguobacterium*			OTU_164, OTU_926		OTU_164
Genus *Ferrimonas*					OTU_2238
Genus *Frankia*	OTU_43530				
Genus *Fulvivirga*		OTU_19475			
Genus *Fusobacterium*				OTU_44093	OTU_44093
Genus *Garciella*	OTU_33821				
Genus *Glaciecola*			OTU_17735		OTU_17735, OTU_23420
Genus *Granulicatella*		OTU_2806, OTU_3637			
Genus *Haliangium*			OTU_1565		
Genus *Halomicronema*	OTU_169, OTU_748, OTU_21418, OTU_23662, OTU_29504, OTU_39562, OTU_43191	OTU_748, OTU_21418, OTU_29504, OTU_34682, OTU_44107, OTU_62583		OTU_21418, OTU_23662, OTU_29504, OTU_34682	
Genus *Herbaspirillum*			OTU_11487		
Genus *Hyphomicrobium*		OTU_46545			
Genus *Inquilinus*		OTU_70486			OTU_44130, OTU_53914, OTU_58822
Genus *Lactobacillus*			OTU_9		OTU_9, OTU_13, OTU_1003
Genus *Lampropedia*				OTU_20315	
Genus *Leptolyngbya*	OTU_39407, OTU_40001			OTU_32585	
Genus *Leptonema*			OTU_8849		
Genus *Lewinella*			OTU_13286		
Genus *Marinomonas*	OTU_946				
Genus *Moraxella*	OTU_15816			OTU_15816, OTU_16724, OTU_20316, OTU_20322, OTU_21925, OTU_21971	
Genus *Muricauda*	OTU_957		OTU_880, OTU_957, OTU_7598	OTU_589, OTU_957, OTU_7598	

Taxa	DMSO-BB	DMSO-Cr	LL-BB	LL-Cr	PFA
Genus *Nisaea*	OTU_26873			OTU_26873	OTU_26873
Genus *Paracoccus*				OTU_71952	
Genus *Pelomonas*		OTU_28818	OTU_28818	OTU_28818	
Genus *Peptoniphilus*			OTU_22978		
Genus *Phaeobacter*			OTU_63336		
Genus *Photobacterium*					OTU_2228, OTU_3218
Genus *Planctomyces*		OTU_12245			
Genus *Porphyromonas*				OTU_27540	
Genus *Prevotella*	OTU_6343			OTU_6343, OTU_7309	
Genus *Pseudomonas*	OTU_16280	OTU_15232, OTU_19927	OTU_15232, OTU_19071, OTU_19888, OTU_19927, OTU_20648	OTU_15696, OTU_20319, OTU_33629	OTU_15232, OTU_19927
Genus *Pseudoruegeria*	OTU_44674, OTU_63624	OTU_44674		OTU_67172	
Genus *Ralstonia*		OTU_10704			
Genus *Rivularia*			OTU_1568	OTU_1568	
Genus *Roseivirga*		OTU_16590			
Genus *Rubritalea*					OTU_23419
Genus *Ruegeria*					OTU_63604, OTU_66706,
Genus *Ruminococcus*		OTU_33514			
Genus *Salinisphaera*			OTU_6543, OTU_19107, OTU_19115	OTU_6821	
Genus SC3-56		OTU_236	OTU_236, OTU_9114, OTU_15982, OTU_16003, OTU_19141	OTU_236	OTU_236
Genus *Schlegelella*				OTU_20314	
Genus SGUS912	OTU_73, OTU_896, OTU_6055, OTU_15792, OTU_15979, OTU_15987, OTU_16008	OTU_73, OTU_896, OTU_6010, OTU_6055, OTU_6132, OTU_8534, OTU_15792, OTU_15979, OTU_15987, OTU_15998, OTU_16008, OTU_16320	OTU_73, OTU_896, OTU_2796, OTU_6009, OTU_6055, OTU_8534, OTU_11195, OTU_11526, OTU_11563, OTU_11619, OTU_15562, OTU_15786, OTU_15792, OTU_15979, OTU_15984, OTU_15987, OTU_15994, OTU_15998, OTU_16008, OTU_16016, OTU_16038, OTU_16349, OTU_16364, OTU_17412, OTU_18914, OTU_20251, OTU_20574, OTU_23166	OTU_73, OTU_896, OTU_5755, OTU_5771, OTU_6009, OTU_6055, OTU_6132, OTU_8534, OTU_11195, OTU_11526, OTU_11563, OTU_15562, OTU_15786, OTU_15792, OTU_15979, OTU_15984, OTU_15987, OTU_15994, OTU_15998, OTU_16008, OTU_16038, OTU_16349, OTU_19169, OTU_19332, OTU_20251, OTU_21282, OTU_21643	OTU_73, OTU_896, OTU_6055, OTU_8534, OTU_15792, OTU_15979, OTU_15987, OTU_15998, OTU_16008
Genus *Shewanella*					OTU_7254, OTU_7279
Genus SMB53			OTU_45438		OTU_45438
Genus *Sphingomonas*		OTU_44009, OTU_44034			OTU_62969

**Taxa**	**DMSO-BB**	**DMSO-Cr**	**LL-BB**	**LL-Cr**	**PFA**
Genus *Spirochaeta*					OTU_7110
Genus *Staphylococcus*			OTU_1800		
Genus *Streptococcus*	OTU_194, OTU_3059	OTU_194, OTU_1258, OTU_2949, OTU_3059, OTU_3312, OTU_3313, OTU_3314, OTU_4336, OTU_4588, OTU_4610, OTU_4881	OTU_194, OTU_2949, OTU_3059		OTU_3351
Genus *Thalassomonas*					OTU_6065, OTU_14965
Genus *Tenacibaculum*					OTU_12015, OTU_17919, OTU_17926
Genus *Turicibacter*					OTU_38107
Genus vadinHB04		OTU_1845			
Genus *Xenococcus*					
Kingdom Bacteria			OTU_13045		OTU_33386, OTU_34520
Lysinibacillus boronitolerans		OTU_293			
Massilia alkalitolerans		OTU_16361			
Massilia haematophila			OTU_15956		
Methylobacterium mesophilicum				OTU_67462	
Methylotenera mobilis		OTU_16274			
Microbacterium chocolatum				OTU_37411	
Micrococcus luteus	OTU_2030			OTU_2030, OTU_37019	OTU_2030, OTU_37019, OTU_37470
Nautella italica			OTU_67439		
Neisseria bacilliformis				OTU_8585, OTU_8588	
Neisseria subflava		OTU_6574, OTU_9257			
Order Burkholderiales			OTU_16039		
Order Chroococcales	OTU_60114				
Order Clostridiales			OTU_18895, OTU_31315	OTU_22705, OTU_29027	
Order Entomoplasmatales			OTU_18908	OTU_18908, OTU_24457	
Order Flavobacteriales			OTU_24408	OTU_24408, OTU_24467, OTU_28814	
Order Gemellales	OTU_3309, OTU_3325, OTU_4356, OTU_6137	OTU_2171, OTU_3065, OTU_4724, OTU_6137	OTU_2171, OTU_3065, OTU_4541, OTU_5012, OTU_6137		OTU_3325, OTU_5062
Order Kiloniellales	OTU_256, OTU_2053, OTU_22449, OTU_25250, OTU_44796, OTU_56905	OTU_256	OTU_256, OTU_1204, OTU_2053, OTU_2854, OTU_22449, OTU_25250, OTU_29130, OTU_29131, OTU_32433, OTU_32484, OTU_33712, OTU_43582,	OTU_256, OTU_1204, OTU_33712	OTU_256, OTU_1204, OTU_1531, OTU_2053, OTU_2854, OTU_18287, OTU_18315, OTU_21723, OTU_22449, OTU_23390, OTU_25250, OTU_27638, OTU_27825, OTU_28514,

Taxa	DMSO-BB	DMSO-Cr	LL-BB	LL-Cr	PFA
			OTU_43584, OTU_43589, OTU_43919		OTU_28515, OTU_28516, OTU_28995, OTU_29007, OTU_29130, OTU_29131, OTU_29132, OTU_29194, OTU_30944, OTU_32000, OTU_32006, OTU_32174, OTU_32178, OTU_32429, OTU_32433, OTU_32484, OTU_32873, OTU_32876, OTU_32877, OTU_34524, OTU_36049, OTU_36685, OTU_43582, OTU_43584, OTU_43589, OTU_43591, OTU_43609, OTU_43815, OTU_43818, OTU_43823, OTU_43919, OTU_43923, OTU_43925 ,OTU_44181, OTU_44188, OTU_49659
Order Legionellales			OTU_16041		
Order Myxococcales		OTU_2260, OTU_6239	OTU_2770, OTU_6239, OTU_6366, OTU_17310	OTU_19, OTU_2770, OTU_3457, OTU_5868, OTU_6239, OTU_6282, OTU_6302, OTU_6512, OTU_7168, OTU_16464 OTU_16899, OTU_22422	
Order Oceanospirillales			OTU_214, OTU_340, OTU_499, OTU_596	OTU_214, OTU_499, OTU_596, OTU_12700	OTU_5804, OTU_6331, OTU_7887, OTU_7890, OTU_7891, OTU_7897, OTU_7900, OTU_7902, OTU_7905, OTU_7908, OTU_8118
Order Phycisphaerales			OTU_25186		
Order RF39				OTU_4	
Order Rhizobiales		OTU_27728, OTU_43799, OTU_44232, OTU_44663	OTU_48912	OTU_44232, OTU_59120, OTU_65671, OTU_66673, OTU_66681	OTU_44232, OTU_66667
Order Rhodospirillales				OTU_2347	OTU_11309, OTU_19828
Order Rickettsiales				OTU_47971, OTU_58188	
Order Roseiflexales	OTU_617	OTU_617		OTU_617	OTU_617
Order Sphingomonadales			OTU_32801	OTU_32801	
Order Vibrionales					OTU_17916, OTU_19379
Paenibacillus barengoltzii	OTU_2256				
Photobacterium damselae					OTU_3083, OTU_3213

Taxa	DMSO-BB	DMSO-Cr	LL-BB	LL-Cr	PFA
Photobacterium rosenbergii	OTU_2052				
Phylum Bacteroidetes		OTU_7566	OTU_7566, OTU_18917, OTU_22021, OTU_22692, OTU_24444, OTU_25190	OTU_7566, OTU_15782, OTU_18917, OTU_22021, OTU_22692, OTU_22693, OTU_24444, OTU_24505, OTU_25190	OTU_7566, OTU_18917
Phylum Spirochaetes					OTU_38106
Phylum SR1				OTU_33993, OTU_35595	
Propionibacterium acnes	OTU_5472, OTU_29486, OTU_32607, OTU_33911, OTU_33913, OTU_34038, OTU_34191	OTU_2942, OTU_5472, OTU_12964, OTU_15337, OTU_15466, OTU_29486, OTU_31721, OTU_32607, OTU_33702, OTU_33906, OTU_33911, OTU_33913, OTU_33935, OTU_33942, OTU_34029, OTU_34038, OTU_34191, OTU_34233, OTU_34236, OTU_34654, OTU_43531	OTU_2942, OTU_5472, OTU_12964, OTU_15337, OTU_15345, OTU_15466, OTU_29486, OTU_31721, OTU_32607, OTU_32890, OTU_33513, OTU_33702, OTU_33906, OTU_33911, OTU_33912, OTU_33913, OTU_33935, OTU_33942, OTU_34029, OTU_34038, OTU_34188, OTU_34191, OTU_34225, OTU_34233, OTU_34236, OTU_34654, OTU_34863, OTU_35438, OTU_36466, OTU_43531	OTU_5472, OTU_15466, OTU_29486, OTU_32607, OTU_33702, OTU_33906, OTU_33911, OTU_33913, OTU_33942, OTU_34038, OTU_34191, OTU_34233, OTU_34236	OTU_2942, OTU_5472, OTU_12964, OTU_15337, OTU_26885, OTU_29486, OTU_32607, OTU_33702, OTU_33911, OTU_33913, OTU_33935, OTU_33942, OTU_34029, OTU_34038, OTU_34188, OTU_34191, OTU_34233
Propionibacterium granulosum		OTU_35447	OTU_35447		
Pseudomonas fragi		OTU_15598	OTU_15598		OTU_15598
Pseudomonas stutzeri				OTU_15254	
Pseudomonas veronii	OTU_7093	OTU_910, OTU_7093, OTU_19203, OTU_19833	OTU_7093, OTU_19203, OTU_19833	OTU_7093	OTU_910, OTU_7093, OTU_15922, OTU_19203, OTU_19833
Pseudoxanthomonas mexicana				OTU_8590	
Roseomonas aerilata		OTU_58103			
Ruminococcus gnavus				OTU_3491	
Spirochaeta halophila	OTU_9094			OTU_9094	OTU_9094, OTU_11465, OTU_11912, OTU_11917, OTU_11939, OTU_12017
Staphylococcus epidermidis	OTU_2781	OTU_2781	OTU_2781, OTU_3780		OTU_2175, OTU_2781, OTU_2945, OTU_3279, OTU_4637, OTU_4760
Stenotrophomonas geniculata	OTU_5826	OTU_5826	OTU_5826	OTU_5826, OTU_8385, OTU_10466	
Veillonella dispar		OTU_753	OTU_753		
Vibrio harveyi					OTU_3210
Xanthobacillum maris				OTU_16726	

C) Top 10 dominant phylotypes - *G. edwardsi*

Taxa	DMSO-BB	DMSO-Cr	LL-BB	LL-Cr	PFA
Anoxybacillus kestanbolensis		OTU_1211, OTU_1223, OTU_1300			OTU_1211, OTU_1223, OTU_1300, OTU_2847
Family Aerococcaceae			OTU_1854		
Family Endozoicimonaceae			OTU_54	OTU_54, OTU_300, OTU_1793	
Family Flammeovirgaceae	OTU_17, OTU_5773	OTU_17			
Family Flavobacteriaceae				OTU_11206	
Family Neisseriaceae				OTU_6489	
Genus *Bacillus*					OTU_3193
Genus *Diaphorobacter*	OTU_3474	OTU_3474		OTU_3474	
Genus *Halomicronema*	OTU_43191				
Genus SC3-56			OTU_236		
Genus SGUS912	OTU_73	OTU_73	OTU_73, OTU_896, OTU_6055	OTU_73, OTU_6055	OTU_73
Genus *Streptococcus*	OTU_194	OTU_194			
Order Kiloniellales	OTU_256		OTU_256		OTU_256
Order Oceanospirillales					OTU_6331
Phylum Bacteroidetes				OTU_7566	
Propionibacterium acnes	OTU_5472	OTU_5472, OTU_29486	OTU_5472, OTU_29486	OTU_5472	OTU_5472
Pseudomonas veronii	OTU_7093	OTU_7093	OTU_7093		OTU_7093
Staphylococcus epidermidis	OTU_2781				

Supp. Table 15. Taxonomic identification of OTUs part of the Core 100% (A), dominant phylotypes (relative abundance ≥ 0.1, B) and top 10 dominant phylotypes (C) in *I. palifera* bacterial assemblage. OTU: Operational Taxonomic Units.

A) Core 100% - *I. palifera*

Taxa	DMSO-BB	DMSO-Cr	LL-BB	LL-Cr	PFA
Anoxybacillus kestanbolensis		OTU_1300			OTU_1300
Brevundimonas diminuta		OTU_67153			
Endozoicomonas montiporae				OTU_2218, OTU_2328, OTU_2342, OTU_2457, OTU_2502	OTU_2218, OTU_2328, OTU_2342, OTU_2434, OTU_2435, OTU_2457, OTU_2465, OTU_2487, OTU_2502, OTU_2536, OTU_2538, OTU_2566, OTU_2573, OTU_2585, OTU_2606, OTU_7199
Family Aerococcaceae	OTU_1854	OTU_2715		OTU_10, OTU_1854	
Family Endozoicimonaceae		OTU_207	OTU_207, OTU_1775	OTU_207, OTU_265, OTU_1087, OTU_1399, OTU_1439, OTU_1587, OTU_1602, OTU_1775, OTU_1799, OTU_1957, OTU_2286, OTU_2411, OTU_2422, OTU_2432, OTU_2445, OTU_2479, OTU_2501, OTU_2523, OTU_2526, OTU_2529, OTU_2540, OTU_2575, OTU_2607, OTU_2623, OTU_2627	OTU_54, OTU_173, OTU_187, OTU_207, OTU_264, OTU_265, OTU_287, OTU_904, OTU_1087, OTU_1399, OTU_1439, OTU_1447, OTU_1480, OTU_1587, OTU_1602, OTU_1775, OTU_1777, OTU_1778, OTU_1784, OTU_1786, OTU_1798, OTU_1799, OTU_1802, OTU_1922, OTU_1957, OTU_2226, OTU_2286, OTU_2372, OTU_2411, OTU_2416, OTU_2419, OTU_2422, OTU_2427, OTU_2432, OTU_2440, OTU_2445, OTU_2460, OTU_2466, OTU_2467, OTU_2468, OTU_2470, OTU_2479, OTU_2481, OTU_2486, OTU_2490, OTU_2495, OTU_2501, OTU_2507, OTU_2522, OTU_2523, OTU_2524,

Taxa	DMSO-BB	DMSO-Cr	LL-BB	LL-Cr	PFA
					OTU_2526, OTU_2529, OTU_2540, OTU_2544, OTU_2545, OTU_2549, OTU_2553, OTU_2567, OTU_2568, OTU_2575, OTU_2578, OTU_2579, OTU_2586, OTU_2605, OTU_2607, OTU_2614, OTU_2623, OTU_2626, OTU_2627, OTU_2636, OTU_2639, OTU_2643, OTU_2646, OTU_2648, OTU_2819, OTU_6534, OTU_6604, OTU_7185, OTU_7197, OTU_7209, OTU_7213, OTU_7239, OTU_7770, OTU_7781, OTU_7811, OTU_9317, OTU_9366, OTU_9384, OTU_11763, OTU_14003
Family Methylobacteriaceae		OTU_15477			
Family Phyllobacteriaceae					OTU_284
Family Ruminococcaceae					OTU_933, OTU_5732
Genus *Bacteroides*		OTU_6168			OTU_9499, OTU_12526
Genus *Delftia*				OTU_18355	
Genus *Diaphorobacter*	OTU_3474, OTU_18563	OTU_969, OTU_3474, OTU_13531, OTU_18563, OTU_19565	OTU_969, OTU_3474, OTU_18563	OTU_969, OTU_3474	OTU_3474
Genus *Klebsiella*		OTU_7973			
Genus *Lactobacillus*					OTU_13, OTU_66, OTU_152, OTU_273
Genus *Marinomonas*					OTU_268
Genus *Parabacteroides*					OTU_8387
Genus *Pseudomonas*			OTU_15232		
Genus *Reinekea*					OTU_2658
Genus SGUS912					OTU_73
Genus *Sphingobium*	OTU_28287				
Genus *Streptococcus*			OTU_194		
Order Clostridiales					OTU_31, OTU_1876
Order Entomoplasmatales					OTU_1641
Order Kiloniellales					OTU_256

Taxa	DMSO-BB	DMSO-Cr	LL-BB	LL-Cr	PFA
Order Myxococcales				OTU_6302, OTU_6539	OTU_237, OTU_239, OTU_6302, OTU_6539
Propionibacterium acnes	OTU_5472, OTU_11342, OTU_29486, OTU_32607, OTU_33702, OTU_33906, OTU_33912, OTU_33935, OTU_33942, OTU_34029, OTU_34038, OTU_34191, OTU_34233, OTU_34236, OTU_43531	OTU_5472, OTU_15337, OTU_29486, OTU_32607, OTU_33911, OTU_33913, OTU_34029, OTU_34038, OTU_34191	OTU_5472, OTU_33913, OTU_34191	OTU_5472, OTU_33911, OTU_33935	OTU_5472, OTU_33913
Pseudomonas fragi			OTU_15598		
Pseudomonas veronii	OTU_7093		OTU_7093, OTU_19203	OTU_7093, OTU_19203	OTU_7093, OTU_19203
Staphylococcus epidermidis	OTU_2781	OTU_2781, OTU_3064			

B) Dominant - *I. palifera*

Taxa	DMSO-BB	DMSO-Cr	LL-BB	LL-Cr	PFA
Acinetobacter lwoffii	OTU_5687, OTU_10899, OTU_10903		OTU_5687		
Bacillus agaradhaerens	OTU_198				
Brevundimonas diminuta		OTU_23567, OTU_59723			
Class Bacilli	OTU_78161				
Escherichia coli			OTU_507		
Family Aerococcaceae	OTU_10, OTU_786, OTU_1854, OTU_2369, OTU_2718, OTU_3060, OTU_4731	OTU_10, OTU_786, OTU_1854, OTU_2369, OTU_2715, OTU_2750, OTU_2944, OTU_3060	OTU_10, OTU_786, OTU_1854, OTU_2369, OTU_2944, OTU_3060	OTU_10, OTU_187, OTU_194, OTU_205, OTU_207, OTU_239, OTU_265, OTU_287, OTU_904, OTU_969, OTU_1087, OTU_1399, OTU_1439, OTU_1587, OTU_1602, OTU_1641, OTU_1775, OTU_1777, OTU_1799, OTU_1854, OTU_1957, OTU_2030, OTU_2175, OTU_2218, OTU_2286, OTU_2294, OTU_2328, OTU_2342, OTU_2411 OTU_2422, OTU_2432, OTU_2435, OTU_2445,	OTU_13, OTU_31, OTU_187, OTU_207, OTU_226, OTU_239, OTU_264, OTU_265, OTU_273, OTU_287, OTU_904, OTU_1009, OTU_1081, OTU_1087, OTU_1284, OTU_1286, OTU_1395, OTU_1399, OTU_1423, OTU_1439, OTU_1440, OTU_1448, OTU_1463, OTU_1602, OTU_1641, OTU_1775, OTU_1777, OTU_1778, OTU_1798, OTU_1799, OTU_1800, OTU_1802, OTU_1876,

Taxa	DMSO-BB	DMSO-Cr	LL-BB	LL-Cr	PFA
				OTU_2457, OTU_2479, OTU_2501, OTU_2502, OTU_2523, OTU_2524, OTU_2526, OTU_2529, OTU_2540, OTU_2573, OTU_2575, OTU_2607, OTU_2614, OTU_2623, OTU_2627, OTU_2648, OTU_2750, OTU_2781, OTU_2944, OTU_3474, OTU_3793, OTU_5472, OTU_5998, OTU_6302, OTU_6481, OTU_6539, OTU_6575, OTU_7093, OTU_7095, OTU_7168, OTU_9207, OTU_12964, OTU_15232, OTU_15598, OTU_17706, OTU_18355, OTU_18563, OTU_19203, OTU_19313, OTU_19600, OTU_19833, OTU_21605, OTU_21606, OTU_22509, OTU_23229, OTU_23967, OTU_24995, OTU_26885, OTU_28287, OTU_28818, OTU_29486, OTU_32268, OTU_32607, OTU_32890, OTU_33702, OTU_33744, OTU_33906, OTU_33911, OTU_33913, OTU_33935, OTU_34029, OTU_34038, OTU_34191, OTU_34233, OTU_34477, OTU_34665, OTU_44527, OTU_45686, OTU_47320, OTU_47497, OTU_59064, OTU_63506	OTU_1922, OTU_1957, OTU_2218O, TU_2226, OTU_2286, OTU_2328, OTU_2342, OTU_2411, OTU_2416, OTU_2422, OTU_2432, OTU_2434, OTU_2435 OTU_2445, OTU_2457, OTU_2479, OTU_2490, OTU_2495, OTU_2501, OTU_2502, OTU_2507, OTU_2523, OTU_2524, OTU_2526, OTU_2529, OTU_2540, OTU_2553, OTU_2567, OTU_2573, OTU_2575, OTU_2578, OTU_2605, OTU_2607, OTU_2614, OTU_2623, OTU_2627, OTU_2636, OTU_2639, OTU_2648, OTU_5472, OTU_5732, OTU_6302, OTU_6534, OTU_6539, OTU_6604, OTU_7093, OTU_9499, OTU_17706
Family Bifidobacteriaceae	OTU_37174				
Family Endozoicimonaceae	OTU_207	OTU_207, OTU_287	OTU_207, OTU_1775		
Family Halomonadaceae		OTU_2294			
Family Methylobacteriaceae		OTU_15477, OTU_50253			
Family Oxalobacteraceae	OTU_23821	OTU_15600			
Family Planococcaceae	OTU_4257				
Family Porphyromonadaceae	OTU_6666				

**Taxa**	**DMSO-BB**	**DMSO-Cr**	**LL-BB**	**LL-Cr**	**PFA**
Family Propionibacteriaceae	OTU_22509, OTU_39510				
Family Salinisphaeraceae	OTU_34146				
Genus *Acinetobacter*	OTU_16417, OTU_32647	OTU_10931	OTU_6019		
Genus *Bacillus*	OTU_3405, OTU_5688		OTU_2394		
Genus *Bacteroides*		OTU_6168	OTU_6168, OTU_7465		
Genus *Bifidobacterium*	OTU_32201				
Genus *Cloacibacterium*	OTU_970, OTU_1513, OTU_22145	OTU_22145, OTU_12410	OTU_970		
Genus *Coprococcus*	OTU_30073				
Genus *Corynebacterium*	OTU_6147, OTU_6790, OTU_12410, OTU_16057, OTU_30862, OTU_33744		OTU_12410, OTU_30862, OTU_33744		
Genus *Delftia*	OTU_18355, OTU_23836	OTU_18355			
Genus *Diaphorobacter*	OTU_969, OTU_3474, OTU_13531, OTU_18563, OTU_19565, OTU_19601	OTU_3474, OTU_18563, OTU_19565	OTU_3474, OTU_18563, OTU_19565		
Genus *Exiguobacterium*			OTU_164		
Genus *Finegoldia*	OTU_28291, OTU_28300, OTU_31095				
Genus *Granulicatella*			OTU_2806, OTU_3637		
Genus *Halomicronema*		OTU_43191			
Genus *Hydrogenophaga*	OTU_25858				
Genus *Janthinobacterium*	OTU_24708				
Genus KD1-23	OTU_21877				
Genus *Klebsiella*		OTU_7973			
Genus *Lactobacillus*			OTU_13		
Genus *Lactococcus*			OTU_3012		
Genus *Massilia*	OTU_22484, OTU_25857				
Genus *Prevotella*	OTU_2717, OTU_31030	OTU_2717			
Genus *Pseudomonas*	OTU_15770, OTU_16293		OTU_15232		
Genus *Pseudoruegeria*		OTU_44674			
Genus *Ralstonia*		OTU_6575			
Genus *Rubrobacter*	OTU_7066				
Genus *Salinisphaera*		OTU_6821			
Genus *Sphingobium*	OTU_28287	OTU_28287			
Genus *Staphylococcus*			OTU_1800		
Genus *Stenotrophomonas*		OTU_6113			
Genus *Streptococcus*	OTU_194	OTU_194, OTU_3059	OTU_194, OTU_1274		
Haemophilus parainfluenzae	OTU_6570				
Micrococcus luteus	OTU_2030, OTU_44076				
Order Actinomycetales	OTU_46520				
Order Chroococcales			OTU_53053		

Taxa	DMSO-BB	DMSO-Cr	LL-BB	LL-Cr	PFA
Order Clostridiales			OTU_11351		
Order Entomoplasmatales	OTU_1641	OTU_1641, OTU_17706			
Order Myxococcales		OTU_239, OTU_6302, OTU_6539	OTU_6302		
Order Salinisphaerales	OTU_12046				
Propionibacterium acnes	OTU_5472, OTU_11342, OTU_29486, OTU_31721, OTU_32607, OTU_32964, OTU_33513, OTU_33702, OTU_33906, OTU_33911, OTU_33912, OTU_33913, OTU_33935, OTU_33942, OTU_34029, OTU_34038, OTU_34191, OTU_34233, OTU_34236, OTU_34654, OTU_34964, OTU_35482, OTU_36335, OTU_36408, OTU_43531, OTU_44274	OTU_5472, OTU_12964, OTU_15337, OTU_29486, OTU_32607, OTU_33702, OTU_33906, OTU_33911, OTU_33913, OTU_33935, OTU_33942, OTU_34029, OTU_34038, OTU_34191, OTU_34233	OTU_5472, OTU_29486, OTU_32607, OTU_33702, OTU_33906, OTU_33911, OTU_33913, OTU_33935, OTU_33942, OTU_34029, OTU_34191		
Pseudomonas fragi	OTU_15598		OTU_15598		
Pseudomonas mendocina	OTU_21772				
Pseudomonas veronii	OTU_4847, OTU_7093, OTU_16278, OTU_22183, OTU_29222	OTU_7093	OTU_7093, OTU_19203		
Staphylococcus epidermidis	OTU_2781, OTU_3279, OTU_4991	OTU_2781	OTU_2175, OTU_2781		
Stenotrophomonas geniculata	OTU_5826	OTU_5826, OTU_6660			
Veillonella dispar		OTU_753	OTU_753		

C) Top 10 dominant phylotypes - *I. palifera*

Taxa	DMSO-BB	DMSO-Cr	LL-BB	LL-Cr	PFA
Family Aerococcaceae	OTU_970, OTU_1854, OTU_2781, OTU_3474, OTU_5472, OTU_7093, OTU_12410, OTU_18563, OTU_29486, OTU_32607	OTU_194, OTU_1641, OTU_1854, OTU_2781, OTU_2944, OTU_3474, OTU_5472, OTU_5826, OTU_15477, OTU_23567	OTU_207, OTU_970, OTU_2781, OTU_2806, OTU_2944, OTU_3474, OTU_5472, OTU_6168, OTU_7093, OTU_29486	OTU_207, OTU_239, OTU_1641, OTU_1775, OTU_1854, OTU_3474, OTU_5472, OTU_6302, OTU_6539, OTU_7093	OTU_207, OTU_287, OTU_1439, OTU_1602, OTU_1775, OTU_2218, OTU_2411, OTU_2422, OTU_2502, OTU_6302
